# Mediterranean Tomato Landraces Exhibit Genotype‐Specific Transcriptomic Responses to Water Stress

**DOI:** 10.1111/ppl.70696

**Published:** 2025-12-18

**Authors:** Aina Juan‐Cabot, Laura Carrillo, Mateu Fullana‐Pericàs, Jeroni Galmés, Joaquín Medina, Miquel À. Conesa

**Affiliations:** ^1^ Agro‐Environmental and Water Economics Research Institute (INAGEA) – Research Group on Plant Biology Under Mediterranean Conditions (PlantMed) – Dept. Biologia Universitat de les Illes Balears (UIB), Complex Balear de Recerca, Desenvolupament Tecnològic i Innovació Palma Spain; ^2^ Centro de Biotecnología y Genómica de Plantas (CBGP‐INIA) CSIC – Universidad Politécnica de Madrid, Campus de Montegancedo Madrid Spain

**Keywords:** abiotic stress signaling, Mediterranean landraces, root and leaf transcriptomics, Tomato (*Solanum lycopersicum*), water stress

## Abstract

Drought stress is a critical limitation to crop production, particularly in Mediterranean climates. The molecular response of drought adaptation in crops is variable and coordinated in leaves and roots. To understand the molecular basis of drought tolerance in tomato, the transcriptomic responses of two drought‐tolerant Mediterranean landraces (‘de Ramellet’ from Spain and ‘Lucariello’ from Italy) were compared with two commercial varieties (‘Moneymaker’ and ‘Ailsa Craig’). Plants were grown under well‐watered and water stress conditions, performing RNA sequencing in leaf and root tissues. Differential expression analysis denoted that the transcriptomic response to water stress was mostly genotype‐dependent and tissue‐dependent. Only 0.9% of leaf DEGs and 5.1% of root DEGs were shared across all genotypes, including pathways like response to salt, response to heat, and photosynthesis. Commercial genotypes regulated a wide array of metabolic pathways, including plant‐defense, indicative of a response to a general metabolic impairment. In contrast, landraces exhibited a more targeted transcriptional response, specifically triggering drought‐ and stress‐related pathways. The ‘de Ramellet’ landrace exhibited a highly divergent leaf response, characterized by increased expression of osmotic stress‐related genes and heat stress pathways, while ‘Lucariello’ regulated salt transmembrane transporters and antioxidant defenses, particularly in roots. These findings highlight the distinct molecular strategies employed by landraces and commercial cultivars under water stress, emphasizing the potential of Mediterranean landraces as genetic resources for improving drought resilience in tomato breeding programs.

## 1 Introduction

Drought stress is a major agricultural challenge, increasingly worsened by climate change (Gupta et al. 2020). Rising global temperatures and unpredictable rainfall patterns are making droughts more frequent and severe, threatening crop yields and food security (Muluneh 2021). The Mediterranean basin is warming 20% faster than the global average (UNEP 2021; COP29 2024). Hence, some of the most important areas for horticultural crop production, like southern Italy and the Iberian Peninsula, are specially affected hotspots (Lazoglou et al. 2024). Horticultural crops, which account for a significant share of vegetable consumption, heavily rely on irrigation for high yields and are particularly vulnerable to water scarcity (Patanè et al. 2011; Pereira et al. 2012; Cramer et al. 2018).

Tomato ( *Solanum lycopersicum* L.) is the most cultivated fruit worldwide, with 192.3 million tonnes produced in 2023 (FAOSTAT 2025). It plays a crucial role in the global diet, especially in Mediterranean countries, which contribute 20% of worldwide production. Notably, four Mediterranean countries (Turkey, Egypt, Italy, and Spain) rank among the world's top 10 producers (FAOSTAT 2025). As water availability declines due to climate change, in particular in the main producing regions, developing drought‐adapted tomato crops is essential (Cammarano et al. 2020; Fuerst et al. 2025). Additionally, tomato is a key model for fleshy‐fruited plants due to its small genome (~900 Mb), short life cycle, well‐characterized diploid genetics, and extensive ‘omics’ resources (The Tomato Genome Consortium 2012; Chen et al. 2020). Enhancing drought tolerance in tomato will therefore provide valuable insights for improving resilience in other horticultural crops.

Drought response is a highly complex, whole‐plant mechanism that drives phenotypic changes, affecting plant morphology, physiology, and ultimately, crop performance (Comas et al. 2013; Gupta et al. 2020). While biotic stress tolerance is often controlled by a few specific genes, improving abiotic stress resilience is more complex, as it involves multiple genes affecting different plant tissues, such as leaves and roots. Moreover, the strategies plants adopt to cope with drought vary widely depending on the species and the specific drought conditions (e.g., intensity, frequency, and duration). Even among closely related species from contrasting environments, responses can differ significantly—some species may escape drought by adjusting their life cycle, while others develop tolerance mechanisms, leading to distinct molecular responses in leaves and roots (Berger et al. 2016). Such diversity in drought adaptation is evident among wild tomatoes and their relatives (*Solanum* sect. *Lycopersicon*, *Lycopersicoides*, *Juglandifolia*). Species from arid environments like *S. pennellii* tend to escape‐like strategy allowing fast growth in short, favourable periods, having high water‐use efficiency under non‐stressing conditions, fast stomatal closure at early stages of drought stress allowing to maintain high leaf water potential, with low investment in leaves (i.e., low leaf mass per area), and tending to survive long‐term drought by triggering leaf senescence and maintaining only apical meristems still active. On the other side, species from more humid environments like *S. pimpinellifolium* tend to tolerance‐like strategies, with higher investment in leaves and roots, and maintaining higher growth rates than many tomato species from arid environments, which resembles the behaviour in the domesticated tomato (Peralta et al. 2008; Easlon and Richards 2009; Conesa et al. 2017; Muir et al. 2017). In fact, abiotic stress tolerance in tomato has traditionally been enhanced through hybridization with its wild relatives like *S. pennellii* and *S. pimpinellifolium* (Bai and Lindhout 2007; Kamenetzky et al. 2010; Bolger et al. 2014). However, since agricultural environments differ significantly from the extreme conditions tolerated by wild species, breeding for drought tolerance traits requires careful selection to prevent negative pleiotropic effects and ensure coordinated modifications in both root and shoot tissues to avoid counterproductive adaptations. This challenge is particularly relevant for elite tomato cultivars grafted onto specialized rootstocks, as mismatches between the stress‐response in scion and in rootstock can hinder adaptation.

An alternative approach lies in the use of local landraces, which offer an underutilized yet valuable genetic reservoir. Unlike modern cultivars, Mediterranean landraces have been naturally selected over centuries for their ability to thrive under suboptimal conditions, often enduring simultaneous water scarcity and heat stress during the main fruiting period. Given their historical adaptation to harsh climates, these landraces represent a promising resource for breeding that can withstand predicted climate change scenarios (Conesa et al. 2020; Lazaridi et al. 2024). Among them, Mediterranean long shelf‐life (LSL) tomato landraces have been traditionally grown with minimal irrigation, developing specific adaptations to water scarcity. Such adaptations involve higher water‐use efficiency than many modern cultivars under stress conditions. At the leaf level, in Balearic landraces, this is achieved by increasing mesophyll airspace fraction and positioning chloroplasts tightly attached to the plasma membrane. This enhances CO_2_ delivery to the chloroplast when stomata are partially closed and thus, with proportionally lower decrease in net photosynthesis (*A*_N_) as compared to water loss through stomata (stomatal conductance, *g*_s_). Adaptation also involves increased leaf vein density, rendering higher hydraulic conductance (Galmés et al. 2011, 2013; Fullana‐Pericàs et al. 2019). Similar behaviours, added to root adaptations enhancing drought tolerance, have been described in LSL landraces from the Eastern Iberian Peninsula and Southern Italy (Patanè et al. 2016; Fullana‐Pericàs et al. 2017; Guida et al. 2017; Giorio et al. 2018; Conesa et al. 2020; Casals et al. 2021). Furthermore, proline accumulation, a trade‐off between *g*_s_ and ABA increase, and fast activation of ROS detoxification machinery, are among the mechanisms conferring drought tolerance to Italian tomato landraces (Landi et al. 2017; Tamburino et al. 2017; Giorio et al. 2018). These adaptive responses involve the expression of numerous genes controlled by a complex regulatory network, including the perception of stress signals, transmission pathways, and activation of stress‐responsive genes, all of which ultimately adjust the plant's physiological and biochemical processes to cope with stress (Zhu 2016). Therefore, studying the molecular mechanisms underlying their exceptional abiotic stress tolerance is crucial for future crop improvement.

Several transcriptomic studies have been conducted to understand the molecular basis of water stress response in tomato, primarily focusing on leaf tissue (e.g., Iovieno et al. 2016; Liu et al. 2017; Zhou et al. 2019; Diouf et al. 2020; Bian et al. 2021). However, the results remain inconsistent, with only a few key pathways consistently identified, such as those related to abscisic acid (ABA) signaling and oxidative stress response (Ercolano et al. 2014; Iovieno et al. 2016; Yang et al. 2022; Liu et al. 2023; Shu et al. 2024). Moreover, key genes identified in the response to further abiotic stresses like heat seem to be also involved in the drought stress response, especially in cases where both stresses co‐occur. This includes genes of diverse heat shock protein (*HSP*) families, mostly *HSP20* and *HSP70*, and heat stress transcription factors like *HsfA3* and *HsfA6B*, the latter also related to ABA signaling and ABA‐mediated heat response (e.g., Iovieno et al. 2016; Haq et al. 2019; Liu et al. 2023; Shu et al. 2024). In contrast, there has been less focus on root transcriptomics responses to water stress, with limited studies available (Balestrini et al. 2019; Karanja et al. 2021). Some key genes described are related to nitrogen use efficiency and acquisition, and also to root development and architecture, like low‐affinity nitrate transporters (e.g., *NRT1.1*; e.g., Renau‐Morata et al. 2021; Hua et al. 2025). Evidence suggests that root adaptation may be linked primarily to cell wall modifications, but comprehensive studies that consider both leaf and root responses are still rare (Zhang et al. 2019; Pirona et al. 2023). Given that many molecular mechanisms operate across different organs, a holistic, whole‐plant perspective is critical to fully understanding drought tolerance. Furthermore, research on Mediterranean landraces transcriptomics is scarce. The few studies available, such as Landi et al. (2023), show that expression patterns in landraces differ significantly from those in commercial elite cultivars. This indicates that landraces may possess unique drought tolerance mechanisms absent in modern cultivars. Nevertheless, since drought tolerance largely depends on a plant's genetic background, studying different tomato varieties is essential for deciphering genotype‐environment interactions and identifying traits that promote resilience (Patanè et al. 2016; Diouf et al. 2020).

The aim of this study is to compare the response to water stress in two drought‐adapted tomato landraces with different origins in the Mediterranean (‘de Ramellet’ from the Balearic Islands and ‘Lucariello’ from Italy) and two widely studied commercial tomato landraces (‘Moneymaker’ and ‘Ailsa Craig’) through transcriptomic analyses of leaves and roots. It is uncovered that molecular responses varied across genotypes, including within landraces, and are tissue‐specific. Environmental factors influenced stress responses, highlighting their role in shaping adaptation. Non‐tolerant genotypes activated general metabolic and defense pathways, while landraces showed targeted adaptations to water, temperature, and osmotic stress. These findings can guide breeding programs to improve drought tolerance by focusing on key adaptive pathways.

## 2 Materials and Methods

### 2.1 Plant Materials

Four genotypes of tomato ( *Solanum lycopersicum* L.) were evaluated in this study: ‘Moneymaker’ (MM), ‘Ailsa Craig’ (AC), ‘Lucariello’ (LUC), and ‘de Ramellet’ (RAM). These genotypes were selected based on previous characterization of genetic diversity, photosynthetic performance, fruit morphometry, shelf‐life, and abiotic stress resilience (Bota et al. 2014; Conesa et al. 2014; Fullana‐Pericàs et al. 2019). MM (accession T100584 from INRAe, Montfavet) and AC (accession NR1045 from COMAV‐UPV seedbank, Valencia) were selected as commercial cultivars with high yield and low resilience to abiotic stress and were selected as two control genotypes upon which to compare local landraces. Selected landraces have higher resilience to abiotic stresses based on photosynthetic activity and yield. Of those, diversity in the geographical origin within the Mediterranean basin was prioritized, with LUC (accession SR‐34 from UNA‐CNR seedbank, Naples) originating from the Campania region in southern Italy and RAM (accession UIB1‐28 from UIB seedbank, Mallorca) from the Balearic Islands in eastern Spain.

Seeds were sterilized in a 50% bleach solution for 15 min and subsequently washed 4 times with sterile distilled water. They were germinated in chambers for 3 days under a stable temperature of 22°C and 16/8 h (day/night) conditions. Germinated seedlings were transferred to pots of 25 cm diameter and 2 kg of peat substrate.

### 2.2 Experimental Design and Treatments

The experiment was performed in technified greenhouse facilities with artificial light supply and temperature control. Plants were grown individually in 10 L pots at 25°C and 16/8 h (day/night) conditions. After 2 weeks, two irrigation blocks were established with 5 plants of every genotype in each, and a completely randomized plant ordination. A block covered 100% of the evapotranspiration (ETP) needs of the plant (well‐watered treatment, WW), and the other was irrigated with only 50% of the WW water volume (water stress treatment, WS) (Figure S1). Leaf gas‐exchange measurements were conducted to assess plant stress level during establishment of the treatments, and to confirm that the irrigation dose in the WS treatment was reduced enough to promote sustained differences at the leaf level between treatments. Once ensured, 5 weeks after treatment establishment, gas‐exchange was measured again, and immediately after, the measured leaf was sampled for RNA extraction, ensuring gene expression can be related to leaf stress level. Just after leaf sampling, root tissue was also sampled from the same plant for RNA extraction. Unearthed root tissue was immediately washed with water under the tap, excess water removed by contact with filter paper, and was flash frozen in liquid nitrogen.

### 2.3 Leaf Gas‐Exchange

**FIGURE 1:**
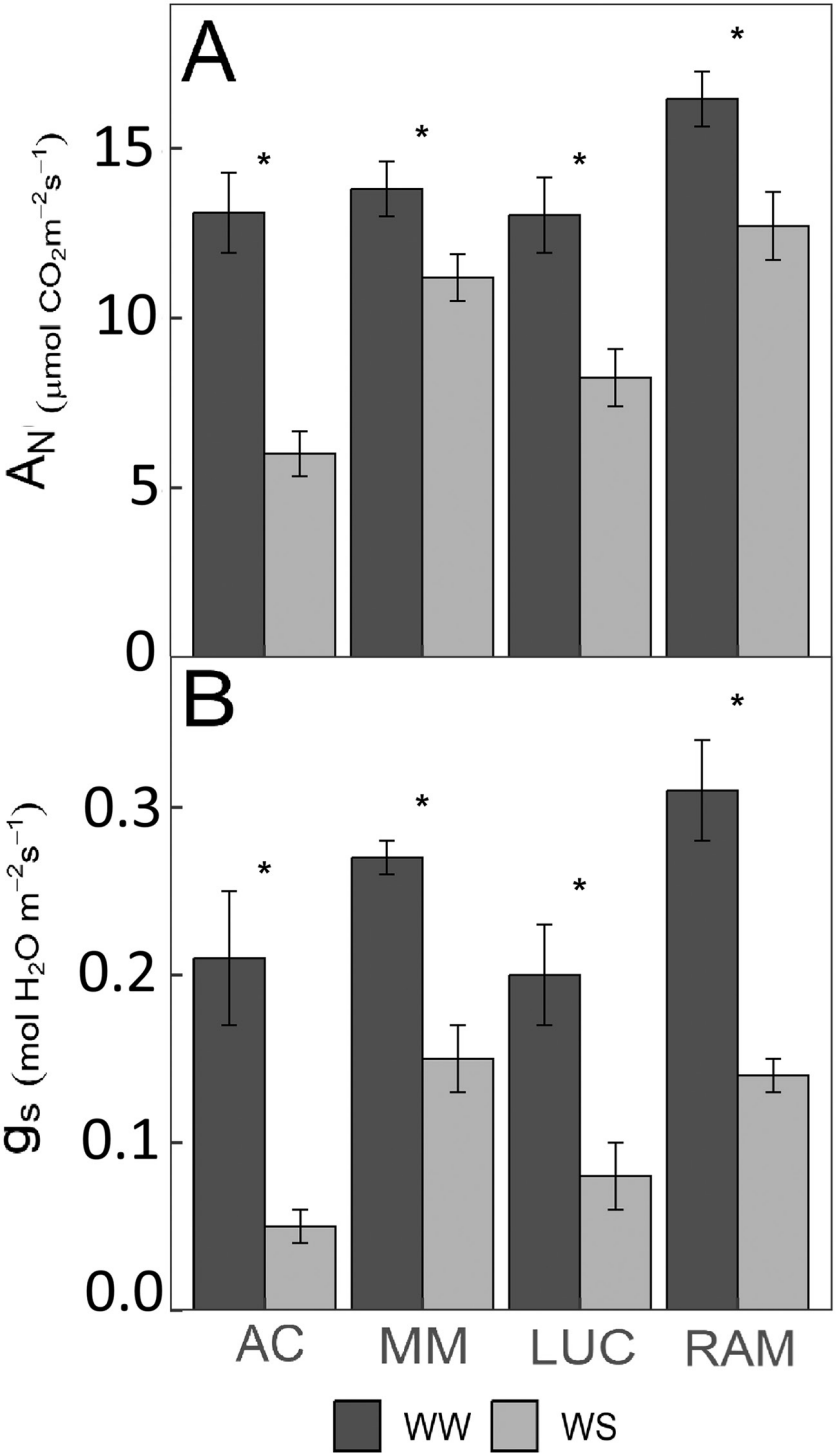
Impact of the drought stress treatment (well‐watered, WW; water stress, WS) in (A) net CO_2_ assimilation rate (*A*_N_) and (B) stomatal conductance (*g*_s_) and in the studied genotypes, ‘Ailsa Craig’ (AC), ‘Moneymaker’ (MM), ‘Lucariello’ (LUC) and ‘de Ramellet’ (RAM) after 5 weeks of water treatments. Bars are averages and error bars represent the standard error (*n* = 4–5). Asterisks denote significant differences between treatments for each genotype (*p*‐value ≤ 0.05).

Leaf gas‐exchange measurements were used only to ensure proper water treatment application, and to demonstrate that there were consistent and significant differences between water treatments in all the genotypes studied at the moment of sampling leaf and root tissue for transcriptomics analyses. To do so, we considered the existence of significant differences in net photosynthesis (*A*_N_) and stomatal conductance (*g*_s_) (Figure 1).

Leaf gas‐exchange was measured with an open infrared gas‐exchange analyzer system (Li‐6400‐40, Li‐Cor Inc.), on five plants per genotype and treatment (one leaf per plant). Measurements were performed in the terminal leaflet of fully expanded leaves, from 09:30 to 13:00. Environmental conditions in the leaf chamber consisted of a photosynthetic photon flux density (PPFD) of 1500 μmol m^−2^ s^−1^ (with 10% blue light), the vapor pressure deficit (VPD) around 1.5 kPa, and a leaf temperature of 25°C. Measurements were performed after inducing steady‐state photosynthesis for at least 30 min at a cuvette CO_2_ concentration (*C*_a_) of 400 μmol CO_2_ mol^−1^ air.

### 2.4 Transcriptomic Analysis

For transcriptomic analyses, we randomly selected three plants per genotype and treatment from the five sampled. The remaining two were kept as a backup. Plant material from leaves and roots was extracted using a RNeasy Plant Mini Kit (Qiagen). For each sample, 1.5 micrograms of DNA‐free RNA were used to generate sequencing libraries by BGI Genomics (BGI Genomics Co. Ltd.). Samples were then sequenced using the BGISEQ platform (BGI Genomics Co. Ltd.). Sequence reads were quality checked using FastQC (www.bioinformatics.babraham.ac.uk/fastqc), and reads were mapped to the NCBI reference genome SL3.0 (https://www.ncbi.nlm.nih.gov/datasets/genome/GCF_000188115.4/) using HISAT software (Kim et al. 2015), considering the annotation in ITAG3.2 genome at SolGenomics when available in order to ease comparison with the literature (https://solgenomics.net/ftp/tomato_genome/annotation/ITAG3.2_release/). Despite the existence of a reference genome for ‘Lucariello’ (Tranchida‐Lombardo et al. 2018), the use of SL3.0 ensured a more comprehensive annotation, allowing standardized comparison with the remaining genotypes used and with data available in the literature.

RNA‐sequencing library preparation, sequencing, and primary data processing (including adapter trimming, quality control, alignment to the reference genome, and generation of raw gene‐level count matrices) were performed by BGI Genomics (BGI Genomics Co. Ltd.). The resulting raw count matrices were used as input for the downstream differential expression analysis described below. See the Data availability section for raw transcript count data access.

Transcriptome variability among samples was assessed through Principal Component Analysis (PCA) using R software (R Core Team). Analysis of variance was performed on the expression level using the transcript abundance (VST‐transformed values of normalized counts) after filtering out genes with low counts (*n* < 10). The contribution of genotype (*G*), environment (*E*), and their interaction (*E* × *G*) to gene expression variation was determined for each gene, employing a two‐way analysis of variance (ANOVA) model; transcript abundance (VST‐transformed values of normalized counts) for each gene was modeled under both treatments. Significant differences were identified using a false discovery rate (FDR) threshold of 5% (Benjamini and Hochberg 1995).

### 2.5 RT‐qPCR Analyses

The total RNA was extracted using the RNeasy Plant Mini Kit (Qiagen), according to the manufacturer's instructions. The gene expression analysis in tomato plants was determined by reverse transcription quantitative PCR (RT‐qPCR) following the procedures described in Corrales et al. (2014). The *UBIQUITIN3* gene from *S. lycopersicum* was used as an internal control for normalization (Hoffman et al. 1991). To validate the RNAseq results, the expression of a group of selected genes was analyzed, considering genes with functional relevance in stress responses and hormone signaling in tomato. The genes used were: low‐affinity *NITRATE TRANSPORTER 1.1* (*NRT1.1*; e.g., Renau‐Morata et al. 2021; Martínez‐Martínez et al. 2024; Hua et al. 2025), *PLASMA MEMBRANE INTRINSIC PROTEIN 2.9* (*PIP2.9*; e.g., Fang et al. 2019; Li et al. 2021), *INDOLE‐3‐ACETIC ACID INDUCIBLE 12* (*IAA12*; e.g., Audran‐Delalande et al. 2012), *HEAT SHOCK PROTEIN 20* and *70* (*HSP20*, *HSP70*; e.g., Haq et al. 2019; Aghaie and Tafreshi 2020; Liu et al. 2023), and *HEAT STRESS TRANSCRIPTION FACTOR 6A* (*A6B*; e.g., Huang et al. 2016; Singh et al. 2018; Shu et al. 2024). A regression model between 2logFC of qPCR relative expression levels and RNA‐seq data for all eight gene models on the four genotypes showed a significant positive correlation between the two analyses (*p*‐value < 0.02; *R* = 0.41) (Figure S2). Therefore, beyond the general validation of the RNAseq dataset, the selection of the aforementioned genes based on their already described key biological relevance allows a robust validation of the impact of the drought stress response in the tomato, according to the previously described. The primer pairs used for amplification are described in Table S4. The relative expression levels of the target genes were calculated by the 2^−ΔΔCT^ method (Livak and Schmittgen 2001). Three plants per genotype and treatment, and three technical replicates per plant (i.e., three aliquots of the same RNA extract) were analyzed.

### 2.6 Statistical Analysis

Two‐way Analysis of Variance (ANOVA) was performed to reveal any effect of the treatment on *A*_N_ and *g*_s_ measurements. For both parameters, one‐way ANOVAs were also performed to reveal differences within each genotype for each treatment (*p*‐value < 0.05 by means of the Tukey test). Similarly, two‐way ANOVAs were performed to reveal the effect of the treatment and genotype on the relative expression of the genes specified in the RT‐qPCR analysis. All statistical analyses were performed using R software (ver. 4.1.0.; R Core Team).

## 3 Results

Leaf gas‐exchange measurements were used only to ensure the existence of consistent differences between well‐watered (WW) and water‐stress (WS) treatments for all the genotypes studied at the moment of sampling leaf and root tissue for transcriptomics analyses, and as an objective reference of the degree of stress applied in the experiment. Leaf gas‐exchange parameters reflected a significant impact of the irrigation treatments, with reduced stomatal conductance (*g*_s_) and net CO_2_ assimilation rate (*A*_N_) in all the genotypes under WS treatment (Figure 1). For *g*_s_, reductions ranged in all genotypes between 46% and 58%, except AC, which suffered a decrease of up to 77%. For *A*_N_, reductions were lowest in MM and RAM (19%–23%), intermediate in LUC (37%), and maximum in AC (54%).

### 3.1 Differential Gene Expression Patterns in Tomato Leaves and Roots Under Water Stress

**FIGURE 2:**
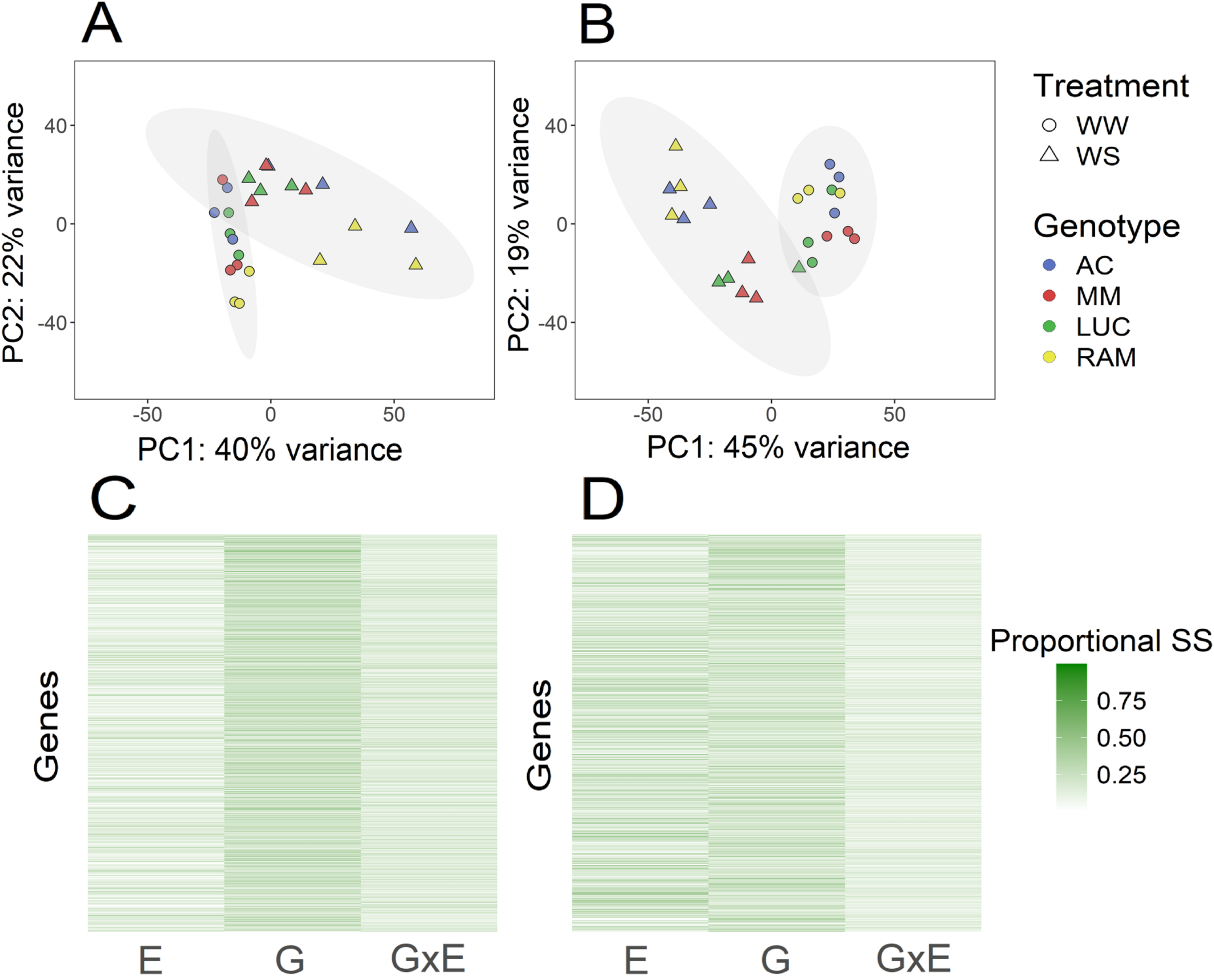
In the top, principal component analyses of the normalized read counts of the RNAseq analysis in leaf (A) and root (B) tissues of ‘Ailsa Craig’ (AC), ‘Moneymaker’ (MM), ‘Lucariello’ (LUC) and ‘de Ramellet’ (RAM) genotypes grown under well‐watered (WW) and water stress (WS) conditions. Three plant replicates per genotype and treatment are represented. The grey ellipses include samples corresponding to the different treatments by superimposing normal‐probability contours on the scatter plot. At the bottom, proportion of the sum of squares attributed to genotype (*G*), irrigation treatment (*E*) and their interaction (*E* × *G*) on the regulation of gene expression in a two‐way analysis of variance (ANOVA) on the VST‐transformed values of normalized transcript levels in leaf (C) and root (D) tissues. Each line represents a single gene from the 23.069 and 23.193 loci studied in leaf and root respectively, and the intensity of green represents the proportion of variation that can be explained by each of the factors.

RNA sequencing was carried out on leaf and root tissues from the four genotypes under the WW and the WS treatments. Up to 22,881 and 23,193 genes with expression levels above background noise were detected in leaf and root tissues, respectively (ca. 66% of the *S. lycopersicum* total Coding Sequence). A PCA with the transformed and normalized read counts revealed that the first two principal components (PC1 and PC2) explained about 63% of the total variability on leaf samples and about 65% on root samples (Figure 2). In both tissues, treatment variability was mostly explained by PC1 (ca. 40% of the total variation), and genotype variability by PC2, the separation being more evident under WS and in root tissue. When considering only leaf tissue, RAM was the most differentiated genotype (Figure 2A). There was a clear separation in PC2 between AC and RAM, especially under WW, which contrasts with the highly similar expression patterns between MM and LUC in both treatments. Results for root tissue (Figure 2B) show larger differences than those observed for leaf tissue, although RAM displayed a very similar behavior to AC in both treatments, and a noticeable separation in PC2 of MM and LUC from AC and RAM was observed under both treatments. Altogether, global expression patterns show higher variation due to the treatment than to the genotype, yet they also allow consistent differentiation of genotypes.

The two‐way analysis of variance (ANOVA) to assess the impact of the environment (*E*; i.e., irrigation treatment), genotype (*G*), and *E* × *G* interaction on the transcriptome reported a total of 12,664 (leaf) and 13,492 (root) genes significantly affected by at least one of the aforementioned factors (*p*‐value ≤ 0.05). In leaves (Figure 2C), gene expression was mainly affected by either the *G* or the *E*, where 34.7% of the genes were significantly affected by *G*, 34.6% by *E*, and 15.1% by the interaction, showcasing the importance of genetic background on gene expression and regulation. In roots (Figure 2D), while *G* is an important factor, gene expression was primarily influenced by *E* (27.6% *G*, 46.2% *E*, 11.2% *E* × *G*).

### 3.2 RNA‐Seq Data Analysis and Evaluation of DEGs in Response to Water Stress

**FIGURE 3:**
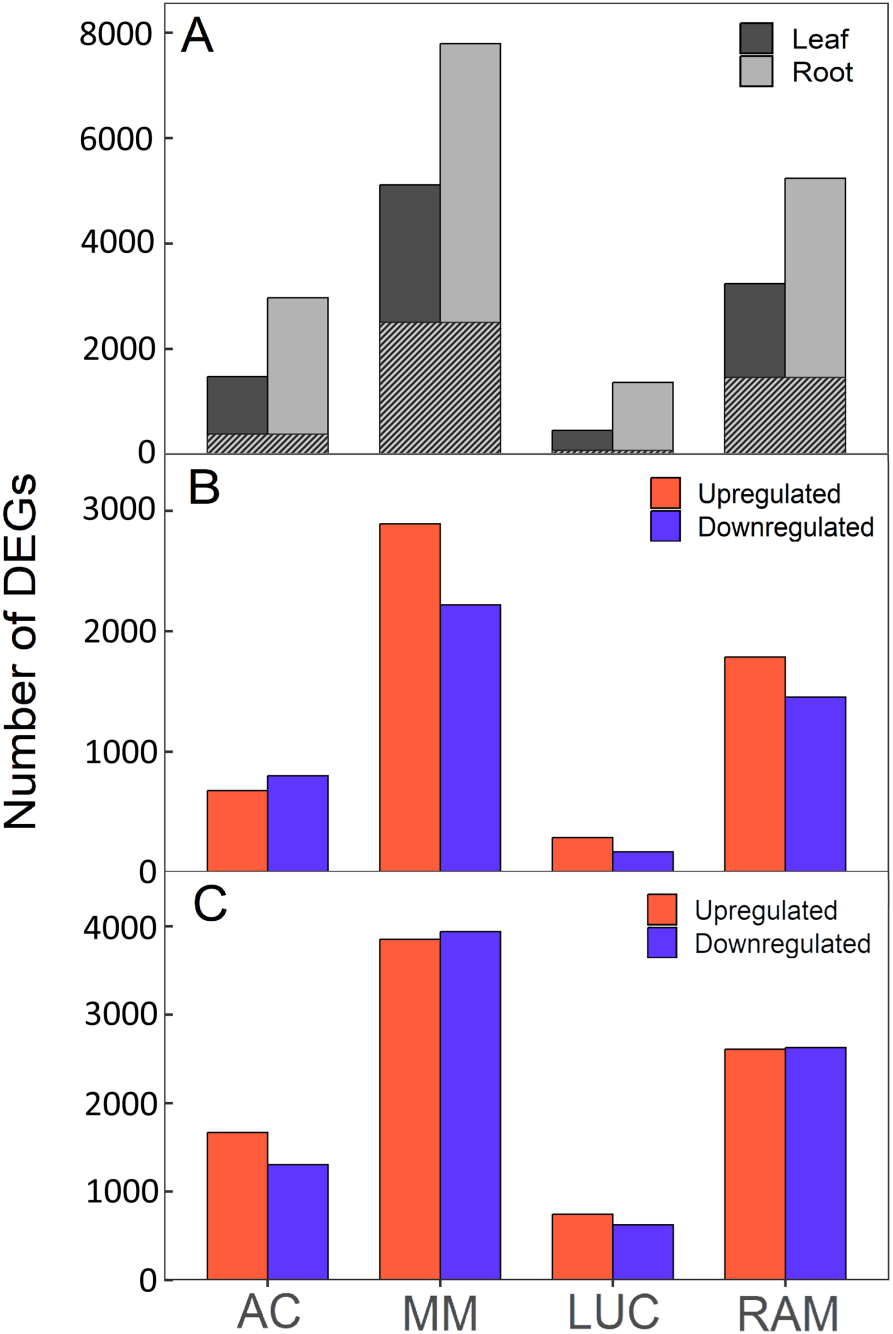
Number of differentially expressed genes (DEGs) in response to water stress per genotype and plant tissue in ‘Ailsa Craig’ (AC), ‘Moneymaker’ (MM), ‘Lucariello’ (LUC), and ‘de Ramellet’ (RAM) genotypes. (A) Number of DEGs exclusive of each genotype in leaves and in roots. The mesh in the columns represents the proportion of such genes that are differentially expressed in both tissues of the same genotype. (B) Number of up‐ and down‐regulated DEGs per genotype in leaves. (C) Number of up‐ and down‐regulated DEGs per genotype in roots.

When considering the impact of the irrigation treatments on gene expression independently for each genotype, from 5107 (MM) to 465 (LUC) differentially expressed genes (DEGs) were identified in leaves, and from 7691 (MM) to 1351 (LUC) in roots (Figure 3A), denoting contrasting responses across genotypes and a larger impact on roots. Moreover, the majority of identified DEGs were organ‐specific in all of them (Figure 3A, mesh vs. non‐mesh pattern). The proportion of up‐ and down‐regulated genes remained consistent across tissues (Figure 3B,C).

In order to understand the global response to drought across genotypes and organs, an analysis without accounting for genotype in the model (i.e., considering together all genes that are DEGs in WS vs. WW in at least one of the genotypes) identified a total of 5248 DEGs in leaves and 3584 in roots (Dataset S1). The lower number of DEGs in roots than in leaves when analyzing all genotypes together, which is opposite to what was observed for each genotype independently (Figure 3A), denotes that the root response is to a large extent common across the studied genotypes, whereas most of the genotype‐specific responses appear at the leaf level. The DEGs considering all genotypes together for both tissues (Dataset S1), and for each specific genotype in leaves (Dataset S2) and in roots (Dataset S3), are detailed in the Supporting Information.

**FIGURE 4:**
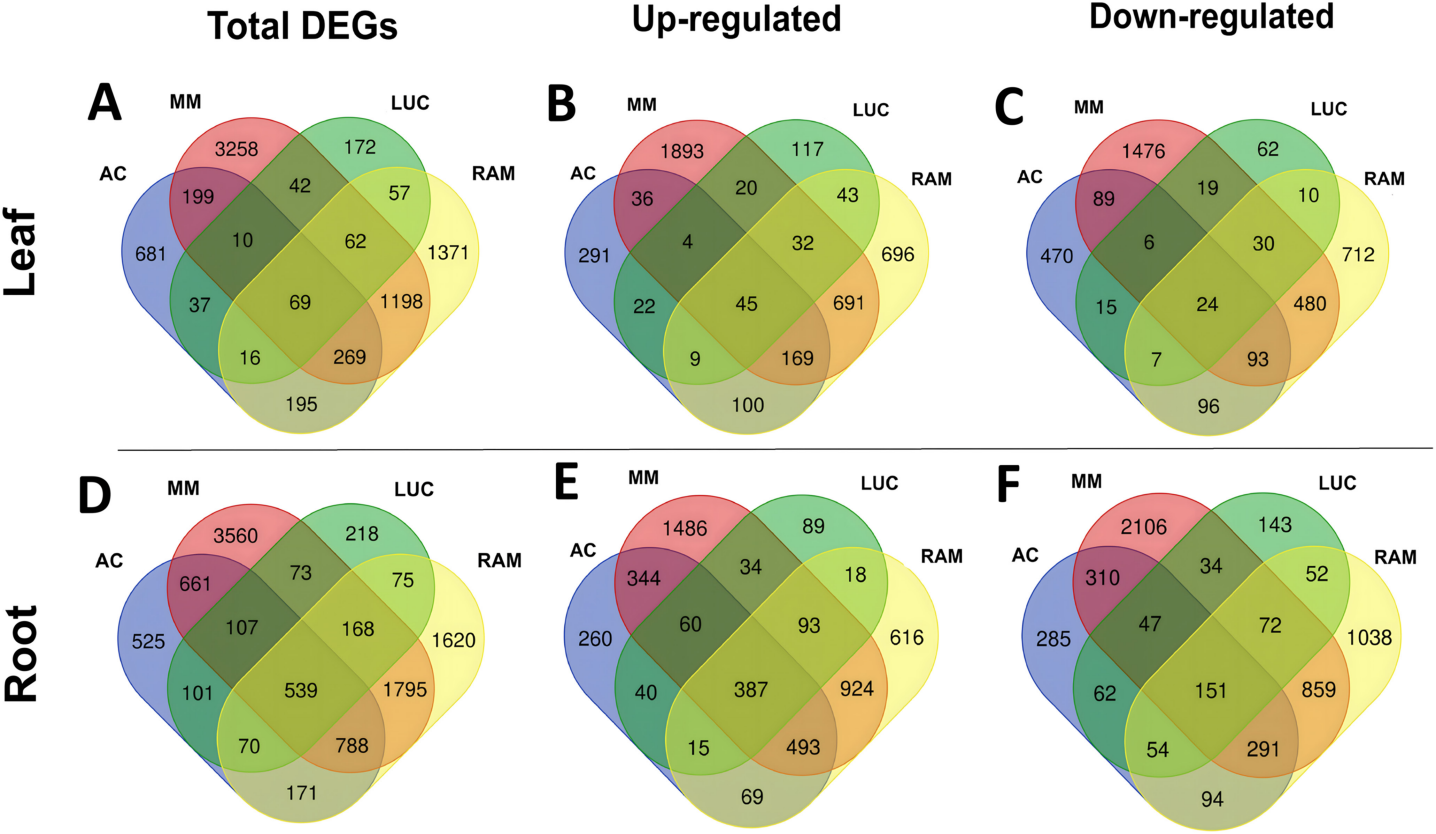
Venn diagrams for leaf (upper row) and root (lower row) tissue, representing all (A, D), the up‐regulated (B, E), and the down‐regulated (C, F) differentially expressed genes (DEGs) detected in ‘Ailsa Craig’ (AC), ‘Moneymaker’ (MM), ‘Lucariello’ (LUC), and ‘de Ramellet’ (RAM) tomato genotypes under water stress when compared to irrigated conditions. Up‐ and down‐regulated gene numbers may be in disagreement with total DEGs due to some genes being up‐regulated in one genotype yet down‐regulated in another, thus causing mismatches. In all cases, the sum for a given genotype in figures B, C, and E, F matches the sum for that genotype in figures A and D, respectively.

On the other hand, the DEGs resulting from independent analyses per genotype are shown for leaves (Figure 4A) and roots (Figure 4D). Three relevant comparisons can be made: the DEGs shared in all genotypes, the DEGs exclusive of landraces, the DEGs exclusive of the controls, and the DEGs exclusive to each genotype. Only 69 DEGs in leaves were shared across all four genotypes, 45 of which were up‐regulated (Figure 4A,B), whereas in roots up to 539 DEGs were shared across all four genotypes, 387 also up‐regulated (Figure 4D,E). In turn, 57 DEGs in leaf and 75 DEGs in root were found to be shared exclusively between LUC and RAM, whereas 199 DEGs in leaf and 661 DEGs in root were shared exclusively between MM and AC (Figure 4A,D). Finally, there were 681, 3258, 172, and 1371 DEGs in leaves exclusive to AC, MM, LUC, and RAM, respectively (Figure 4A); whereas in roots, 525, 3560, 218, and 1620 DEGs were exclusive of AC, MM, LUC, and RAM, respectively (Figure 4D).

### 3.3 Gene Ontology Enrichment Analysis of RNA‐Seq Data

The Gene Ontology (GO) Enrichment Analysis allowed for the identification of genetic pathways involved in the observed DEGs due to the irrigation treatments, considering the three comparisons indicated in the previous section. First, GO analysis of the shared DEGs across all four genotypes revealed various Biological Process GO terms relevant to the shared responses to water stress (Dataset S1). Notably, these terms included the response to heat (GO:0009408, *p*‐value < 10^−9^) and response to salt (GO:1902074, *p*‐value < 10^−4^). However, the most biologically significant GO term was photosynthesis (GO:0015979, *p*‐value < 0.001), with up to 68 DEGs (Dataset S1), of which 27 DEGs specifically associated with the photosynthesis pathway (KEGG:00195; Table S1).

Second, in order to identify similarities and differences between landraces (RAM and LUC) and controls (AC and MM), GO Enrichment Analyses were focused on genes differentially expressed exclusively in both LUC and RAM, and in both AC and MM. Notably, none of the control genotypes showed significant enrichment for key stress‐related GO terms, such as response to water deprivation (GO:0009414) and water transmembrane transporter activity (GO:0005372), both of which were significantly enriched (*p*‐value < 0.05) in the leaves of the two landraces (Dataset S4). In roots, both landraces exhibited significant enrichment for lipid storage (GO:0019915), response to heat (GO:0009408), and salt transmembrane transporter activity (GO:1901702) (Dataset S5). In turn, analyses on genes shared between control genotypes identified several common pathways in the control genotypes' response, including the up‐regulation of the cutin biosynthetic process (GO:0010143), suberin biosynthetic process (GO:0010345), and wax biosynthetic process (GO:0010025) in leaves. In roots, the regulation of mRNA splicing (GO:0048024) was up‐regulated, while endopeptidase inhibitor activity (GO:0004175), cell wall organization (GO:0007047), galacturonan metabolic process (GO:0010393), and pectin metabolic process (GO:0045488) were down‐regulated.

**FIGURE 5:**
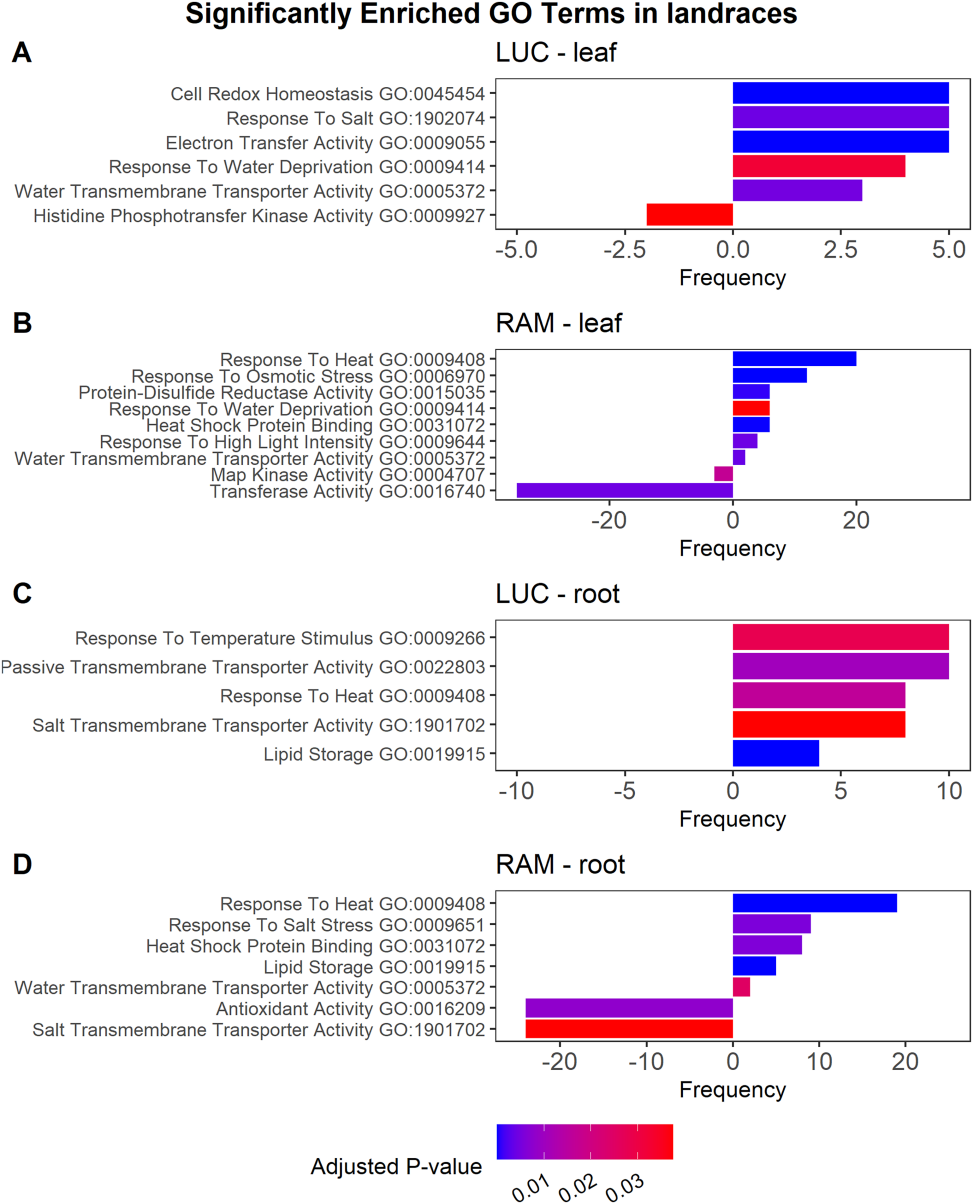
Most significantly enriched Gene Ontology terms in landraces for DEGs detected exclusively in ‘Lucariello’(LUC) (A, C) and ‘de Ramellet’ (RAM) (B, D), in leaves (A, B) and in roots (C, D), via functional profiling. GO categories enriched in up‐regulated DEGs are represented to the right and enriched in down‐regulated DEGs to the left.

Finally, in order to determine the genotype‐specific response to drought, GO Enrichment Analyses were focused on genes related to genotype‐ and tissue‐specific DEGs. For the landraces, in leaves LUC exhibited significant enrichment in terms related to response to salt (GO:0009651), electron transfer activity (GO:0009055), and cell redox homeostasis (GO:0045454), among others (Figure 5A). Conversely, in leaves RAM showed significant enrichment in terms (*p*‐value < 0.05) associated with *HSP* binding (GO:0031072), response to high‐light intensity (GO:0009644), and response to osmotic stress (GO:0006970), among others (Figure 5B). In roots, LUC did not exhibit down‐regulated pathways and showed increased expression of genes related to response to temperature stimulus (GO:0009266) and passive transmembrane transporter activity (GO:0022803) (Figure 5C). In contrast, RAM up‐regulated genes linked to *HSP* binding (GO:0031072), water transmembrane transporter activity (GO:0005372), and response to salt stress (GO:0009651), while downregulating genes associated with antioxidant activity (GO:0016209) (Figure 5D). Interestingly, one of the GO terms common in both landraces in roots, salt transmembrane transporter activity (GO:1901702), was up‐regulated in LUC but down‐regulated in RAM (Figure 5C,D). Notably, this was the only pathway identified with an opposite regulation pattern between the two landrace genotypes. It is also worth noting that, unlike the remaining accessions, in RAM up to three GO terms were up‐regulated both in leaves and in roots (response to heat, GO:0009408; *HSP* binding, GO:0031072; and water transmembrane transporter activity, GO:0005372; Figure 5B,D).

**FIGURE 6:**
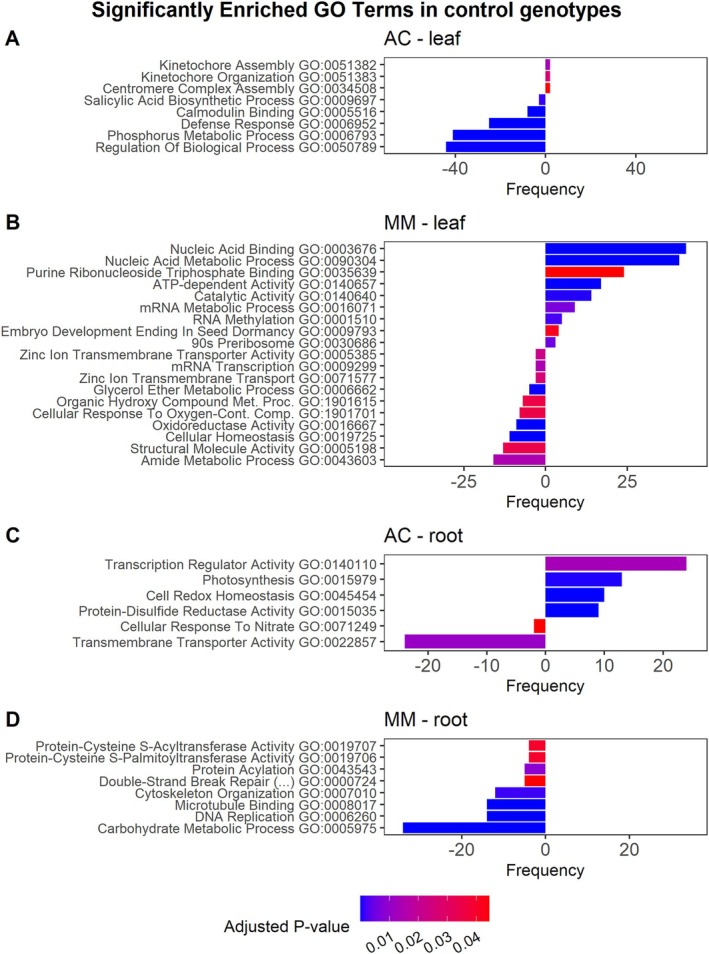
Most significantly enriched Gene Ontology terms in control genotypes for DEGs detected exclusively in ‘Ailsa Craig’ (AC) (A, C) and ‘Moneymaker’ (MM) (B, D), in leaves (A, B) and in roots (C, D), via functional profiling. GO categories enriched in up‐regulated DEGs are represented to the right and enriched in down‐regulated DEGs to the left.

For the controls, in leaves, GO analyses of up‐regulated genes of the AC genotype showed significant enrichment of three GO terms related to chromosome organization (GO:0051276), specifically GO:0051383, GO:0051382, and GO:0034508. GO terms associated with metabolic regulation (GO:0050789, GO:0006793) and defensive pathways (GO:0009697, GO:0006952) were down‐regulated (Figure 6A). MM showed a more complex response, with significant enrichment in nine up‐regulated and 10 down‐regulated GO terms (Figure 6B). The up‐regulated were linked to nucleic acid metabolic processes (GO:0001510, GO:0016071, GO:0090304), and nucleic acid binding and catalytic activity (GO:0003676, GO:0035639, GO:0140640, GO:0140657), whereas the down‐regulated were associated with mRNA transcription (GO:0009299), general metabolic processes (GO:0006662, GO:1901615, GO:0043603), and cellular homeostasis (GO:0019725), among others. In roots, AC showed an enrichment of up‐regulated terms related to cell redox homeostasis (GO:0045454), transcription and catalytic activity (GO:0015035, GO:0140110), and photosynthesis (GO:0015979), whereas down‐regulated genes showed enrichment in transmembrane transporter activity (GO:0022857) and cellular response to nitrate (GO:0071249) (Figure 6C). In contrast, MM did not show significant enrichment in up‐regulated genes of root pathways but exhibited down‐regulation in terms related to cell division (e.g., cytoskeleton organization, microtubule binding, DNA replication, and repair; GO:0007010, GO:0008017, GO:0006260) and primary metabolism of proteins and carbohydrates (GO:0043543, GO:0005975, GO:0019706, GO:0019707) (Figure 6D).

### 3.4 Validation of the Expression Patterns Under Drought Stress Based on Relevant Genes in Tomato

The most biologically relevant DEGs in leaves and roots associated with enriched pathways in the drought‐tolerant landraces are summarized in Tables S2 and S3. Among the genes shared between both landraces, several members of the *HSP* family, particularly *HSP20* and *HSP70*, stood out in both leaf and root tissues of RAM and LUC (Tables S2 and S3). In roots, a notable up‐regulation was observed in genes belonging to the plasma membrane intrinsic proteins (*PIP*) family, specifically *PIP1* and *PIP2*.

**FIGURE 7:**
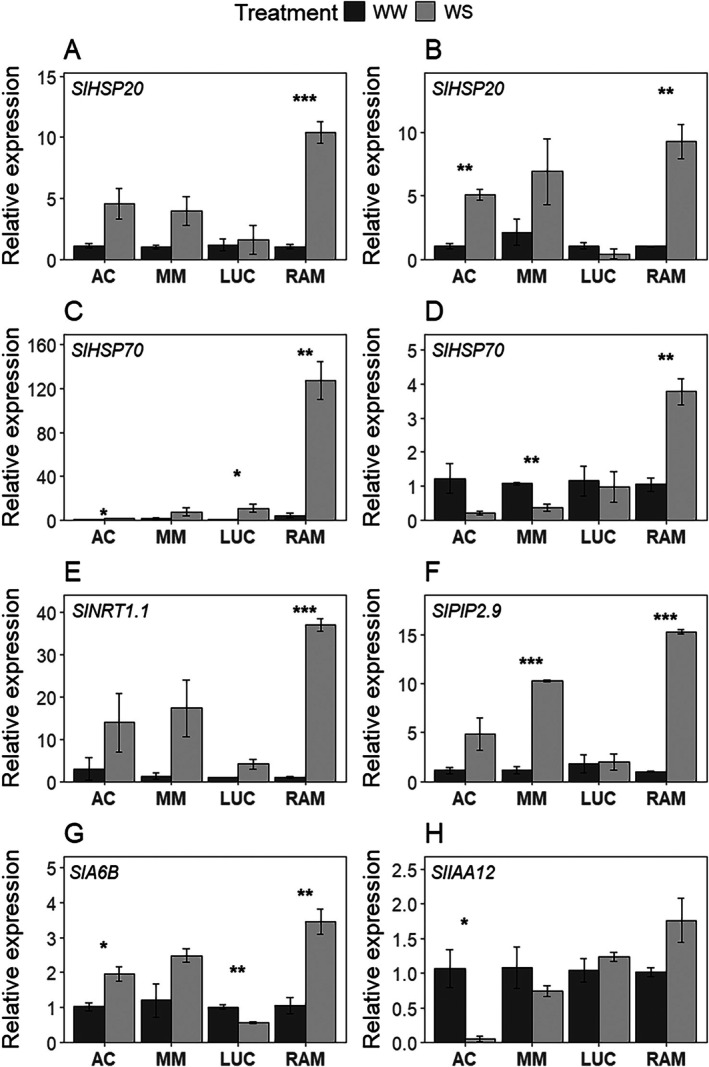
Relative expression of heat shock proteins *HSP20*, *HSP70*, nitrate transporter *NRT1.1*, and heat factor *A6B* in leaf tissue (A, C, E, G), and heat shock proteins *HSP20*, *HSP70*, plasma membrane intrinsic protein *PIP2.9*, and auxin inducible protein *IAA12* in root tissue (B, D, F, H), for ‘Ailsa Craig’ (AC), ‘Moneymaker’ (MM), ‘Lucariello’ (LUC), and ‘de Ramellet’ (RAM) tomato genotypes under control (WW) and water stress (WS) conditions. Asterisks denote statistically significant differences at *p*‐value ≤ 0.05 (*), *p*‐value ≤ 0.01 (**), and *p*‐value ≤ 0.001 (***) by means of Tukey test.

Quantitative analysis via RT‐qPCR confirmed the differential expression between WS and WW in the studied genotypes. The expression of key genes among the highlighted above was investigated: four in leaves (*SlHSP20, SlHSP70, SlNRT1.1*, and *SlA6B*) and four in roots (*SlHSP20, SlHSP70, SlPIP2.9* and *SlIAA12*) (Figure 7). In particular, for the heat‐shock proteins, under WS conditions RAM up‐regulated *SlHSP20* in both tissues (9.9‐fold higher than WW in leaves, *p*‐value < 0.001, and 9.2‐fold higher than WW in roots, *p*‐value < 0.01; Figure 7A,B), while changes were non‐significant in the remaining genotypes, except a 4.8‐fold increase under WS in roots of AC (*p*‐value < 0.01), yet with a very basal expression level (Figure 7B). Similarly, *SlHSP70* expression was significantly more induced by WS in RAM in both tissues (29‐fold higher in leaves, and 3.6‐fold higher in roots, *p*‐value < 0.01 in both cases; Figure 7C,D). In leaves AC (1.7‐fold higher in WS, *p*‐value < 0.05) and LUC (10.7‐fold higher in WS, *p*‐value < 0.05) followed a similar trend, whereas in roots LUC and the controls followed an opposite trend to RAM, tending to decrease *SlHSP70* expression under WS, despite differences being significant only in MM (2.9‐fold higher under WW, *p*‐value < 0.01; Figure 7D).

Similar to the observed for *HSP*s, the nitrate transporter *NRT1.1* was significantly induced in leaves in RAM (35‐fold higher in WS, *p*‐value < 0.001), with a similar trend in the remaining genotypes despite differences between treatments being non‐significant (Figure 7E). Regarding the heat stress factor *SlA6B* in leaves, RAM was also the genotype showing the largest increase under WS (3.3‐fold higher, *p*‐value < 0.01; AC 1.9‐fold higher, *p*‐value < 0.05) and, notoriously, its expression was decreased under WS in LUC (1.8‐fold lower, *p*‐value < 0.01) (Figure 7G). In roots, *SlPIP2.9* was up‐regulated under WS in MM and especially in RAM (9.1‐fold higher and 14.9‐fold higher, respectively, *p*‐value < 0.001 in both cases), with non‐significant differences in AC and LUC, despite the large expression of this gene in the latter genotype under both treatments (Figure 7F). Finally, there was a trend in increasing *SlIAA12* expression under WS in the landraces and in decreasing its expression under WS in the controls, although differences were significant only in AC (20.5‐fold lower in WS, *p*‐value < 0.05; Figure 7H).

## 4 Discussion

To investigate the molecular responses of Mediterranean landraces to water deficit, this study examined two drought‐tolerant LSL tomato landraces from distinct geographic origins: ‘de Ramellet’ (RAM) from the Balearic Islands (Spain) and ‘Lucariello’ (LUC) from Campania (Italy). These were compared with two widely used commercial control varieties, ‘Moneymaker’ (MM) and ‘Ailsa Craig’ (AC). Mediterranean LSL tomato landraces have been maintained on‐farm for centuries, with growers commonly self‐storing their seed every season or every few seasons. This, added to the high levels of self‐pollination in tomato, resulted in local landraces constituting populations of inbred lines (Terzopoulos and Bebeli 2008; Cebolla‐Cornejo et al. 2013; Corrado et al. 2014; Conesa et al. 2020; Villena et al. 2023; Caramante et al. 2024). Thus, their adaptive capacity to stress derives from the presence of diverse inbred lines with variable performance (e.g., Zeven 1998; Casañas et al. 2017). Consequently, seedbank accessions may be heterogeneous, containing mixtures of lines that differ in stress responses.

Plant pooling is a common strategy in genetic and transcriptomic studies to increase the number of plants considered and, especially, to minimize biological variability. However, in this study, RNAseq analyses were performed for each individual plant in order to detect possible genetic variability resulting from heterogeneous seed‐stocks. Results showed that variability among plants of the same accession was low (see Figure 2, and read count data in supplemental information). Alternative studies with the same landrace accessions using plant‐pooled biological replicates or an increased number of plant replicates might reinforce the conclusions in this study.

Given the goal of identifying traits that enhance drought tolerance in agricultural settings, the experimental conditions were designed to reflect realistic water stress scenarios rather than extreme conditions (Gilbert and Medina 2016). This approach ensured proper plant development in early stages while avoiding stress levels that might trigger severe damage pathways in less tolerant genotypes, allowing for a more accurate assessment of the early plant response to stress.

At the physiological level, WS treatment had a significant impact on all the studied genotypes by reducing the stomatal conductance (*g*_s_) and, therefore, the net CO_2_ assimilation rate (*A*_N_) due to decreased leaf internal CO_2_ concentration (Figure 1). Stomatal closure is the prime response at short term in plants under water shortage (Flexas et al. 2006) and has been widely used as a physiological indicator of the severity of stress. In this sense, the decrease in *g*_s_ in RAM, LUC, and MM, of around 50%, is indicative of moderate stress on these genotypes, while the larger decrease in *g*_s_ observed for AC (77%) suggests a larger sensitivity of this genotype to WS treatment. It is worth mentioning that a variable degree of stress across experiments may account for a relevant part of the variation in the results existing in the literature, in particular related to transcriptomics, metabolomics, and biochemistry. In this study, by providing data on *A*_N_ and *g*_s_ at the moment of sampling for transcriptomics (Figure 1), this allows researchers to objectively discuss the degree of stress as responsible for possible discrepancies across studies using the same or similar genotypes.

### 4.1 Leaf and Root‐Specific Responses to Water Stress in Tomato Landraces and Commercial Varieties

Transcriptomic analysis of landraces and commercial varieties revealed significant differences among genotypes under both WW and WS conditions, with the largest differences found under WS (Figure 2). These findings suggest that the transcriptomic profile was highly dependent on the genotype and the treatment. The leaf transcriptomic response to WS was more pronounced in RAM and MM than in LUC and AC, with RAM displaying the most distinct gene expression patterns under WS (Euclidean distance from WW centroid: AC = 29.3, MM = 46.3, LUC = 27.9, RAM = 53.4). This result aligns with previous findings indicating that RAM possesses key morpho‐physiological adaptations for drought tolerance (Galmés et al. 2013). Additionally, transcriptomic differences between RAM and AC compared to LUC and MM extended to root responses, reinforcing the idea that both leaves and roots exhibit distinct, genotype‐dependent drought response mechanisms.

The strong influence of the genetic background on stress responses in tomato has been previously reported (Foolad et al. 2003; Ripoll et al. 2016; Diouf et al. 2020), with environmental conditions and stress severity playing a crucial role in shaping these responses. Interestingly, while some studies suggest that only a minor fraction of the transcriptomic response to drought is genotype‐specific (e.g., in ‘M82’; Pirona et al. 2023), our findings emphasize a significant genotype‐dependent component, particularly in leaf responses, as seen when considering the effects of genotype (*G*), environment (*E*), and their interaction (*G* × *E*) on drought responses (Figure 2). Nevertheless, it is worth mentioning that some part of the differences in the transcriptomic response when comparing among studies may result from the different kinds of stress imposed. The present study considered a permanent drought stress progressively imposed for 4 weeks, resembling common cultivation conditions of the studied landraces outdoors without irrigation (e.g., see Conesa et al. 2020 for details). This differs from short‐term shock treatments (e.g., Pirona et al. 2023) and cycles of drought and rehydration (e.g., Iovieno et al. 2016; Landi et al. 2023) treatments used in other studies and thus must be taken into account when evaluating the differences described among genotypes and the genes involved in the drought stress response.

### 4.2 Landraces Regulate Stress‐Related Pathways to Face Water Stress, While Commercial Varieties Impair General Metabolism and Deploy Plant Defense Pathways

Although key photosynthetic parameters were notably impacted by WS in all genotypes (Figure 1), no significant genotype‐specific DEGs were associated with photosynthesis. This fact suggests that the variations in WS response between landraces and control genotypes are not primarily driven by differences in the regulation of photosynthetic pathways.

In contrast, GO enrichment analysis revealed that pathways related to drought, heat, and salt stress were regulated in a genotype‐ and tissue‐specific manner (Figures 5 and 6). Notably, only the two drought‐tolerant landraces consistently regulated key water stress pathways, including water deprivation response and water transmembrane transporter activity in leaves, as well as salt transmembrane transporter activity in roots. The fact that these pathways remained significant even when excluding common DEGs between landraces from the analyses further suggests that genotype‐specific DEGs contribute to drought adaptation, potentially through distinct molecular mechanisms. Interestingly, lipid storage in roots was consistently regulated under water stress conditions in the landraces but not in the control genotypes. Although this study did not directly identify ABA production and signaling pathways, the described involvement of ABA in root lipid regulation and protein storage (Hossain et al. 2016) suggests a potential role in regulating gene expression, particularly in AC and MM genotypes.

Heat stress response pathways were up‐regulated in roots of both RAM and LUC landraces but not in control genotypes, indicating that landraces activate heat‐stress‐related genes under drought, even without heat stress. This may reflect their evolution under combined summer heat and water stress (Conesa et al. 2020) or suggest that GO‐classified heat stress genes can also function in drought response, at least in certain tomato genotypes (Iovieno et al. 2016; Landi et al. 2023). Notably, HSP70 and HSP20 families were key drought‐responsive genes in landraces, consistent with findings in ‘Moneymaker’ (Shu et al. 2024) but contrasting with other genotypes like ‘M82’ (Iovieno et al. 2016; Liu et al. 2023). These results highlight the genotype dependence of drought responses and suggest that differences in genetic background and stress intensity (permanent vs. short‐term stress) influence HSP activation, reinforcing previous observations in tomato (Diouf et al. 2020; Landi et al. 2023).

Additionally, heat stress response pathways were up‐regulated in roots of both landraces but not in control genotypes, indicating that RAM and LUC landraces activate heat‐stress‐related genes when exposed to drought, even under non‐heat‐stress conditions. This aligns with the idea that Mediterranean landraces, which evolved under extreme summer conditions with combined water and heat stress (Conesa et al. 2020), have developed integrated tolerance mechanisms for both stresses. Alternatively, it suggests that genes classified under heat stress pathways in the GO database may also be involved in drought response, at least in certain tomato genotypes, as observed in other studies (Iovieno et al. 2016; Landi et al. 2023). A well‐known example of this overlap in stress responses is the role of *HSP*s. Various HSP families, including *HSP70* and *HSP20*, have been reported to be up‐regulated under drought, salinity, light, and heavy metal stresses in multiple crops (Haq et al. 2019). In this study, *HSP70* and *HSP20* families were among the key genes involved in drought response in landraces but not in control genotypes (Figure 7; Tables S2 and S3), agreeing with other water stress studies using ‘Moneymaker’ (Shu et al. 2024). Contrarily, *HSPs* were detected in ‘M82’ (Iovieno et al. 2016; Liu et al. 2023), further supporting the idea that drought response in tomato is highly genotype‐dependent. Indeed, the present study identifies the *HSP70* family as a key component of drought tolerance in Mediterranean tomato landraces. Previously, Landi et al. (2023), considering the LUC landrace, did not highlight HSPs as major players in drought response. This discrepancy suggests that both genetic background and stress intensity play a crucial role in triggering stress tolerance mechanisms, further reinforcing previous findings in tomato (Diouf et al. 2020). Since the intensity and duration of the drought stress are different in the present study (i.e., permanent stress progressively applied) and in others (e.g., shock stress in Iovieno et al. 2016; short‐stress and rehydration cycles in Landi et al. 2023), further research is needed to ascertain which part of this response is certainly genotype‐specific or stress‐severity dependent.

LUC and RAM also exhibited significantly higher expression levels of several *Glutaredoxin* genes (*GRXs*), unlike control genotypes (Table S2). *GRX* genes, encoded as small heat‐stable disulfide oxidoreductases, play a key role in antioxidative responses by regulating protein activity through glutathionylation (Meyer et al. 2009; Guo et al. 2010). Interestingly, LUC and RAM shared only two out of the 11 identified *GRX* genes, indicating that while both genotypes activate antioxidative stress responses under WS, they rely on distinct gene sets. Additionally, both landraces showed higher levels of expression of several *Dehydrins (DHNs)* in response to WS in leaves and roots, a pattern consistent with previous findings (Yamasaki et al. 2013; Liu et al. 2015; Iovieno et al. 2016), including landraces (Landi et al. 2023).

In contrast, the control genotypes exhibited a distinct response, primarily regulating genes associated with structural reinforcement and defense‐related pathways, like genes involved in the biosynthesis of cutin, suberin, and wax in the leaves, as well as genes related to cell wall organization in the roots. To our knowledge, this response has not been previously described for AC, although Živanović et al. (2021) reported increased cell‐wall stiffness in this genotype under drought, suggesting that enhanced structural fortification may contribute to its stress adaptation. These metabolic changes, and a generalized metabolic adjustment under drought conditions, are consistent with findings in other non‐tolerant tomato genotypes (Veronico et al. 2022; Xu et al. 2022; Landi et al. 2023; Liu et al. 2023; Pirona et al. 2023).

Additionally, AC and MM were found to influence general plant defense mechanisms and salicylic acid biosynthesis. Salicylic acid, a key stress‐signaling phytohormone—alongside jasmonic acid and ethylene—plays a crucial role in plant defense by reducing stress sensitivity, regulating stomatal closure and transpiration, and activating genes involved in both biotic and abiotic stress responses (Hossain et al. 2016; Saleem et al. 2021). Moreover, Muñoz‐Espinoza et al. (2015) also found that salicylic acid biosynthesis was affected in both AC and MM under drought, while our findings relate to salicylic acid biosynthesis modifications under drought stress for AC. However, none of these pathways appeared to be significantly regulated in response to water stress in the control genotypes. Strikingly, AC also upregulated photosynthesis pathways in roots under stress. This has been observed previously in tomato (Pirona et al. 2023) and rice (Minh‐Thu et al. 2013; Baldoni et al. 2016), and it has been related to opposite expression patterns in leaf and root induced by drought stress.

Validation of the gene expression dataset was performed in this study by using four genes in leaves and four genes in roots (Figure 7). The selected genes have been previously described in tomato (and other crops) as having relevant biological function under variable types of stress. Therefore, in addition to the mere validating function, selected genes allowed a robust validation of the impact of the drought stress applied in this study, agreeing with previous studies in tomato. In particular, such genes included *HSP20* and *HSP70*, which have been described as relevant in a wide array of biotic and abiotic stresses including drought and heat (e.g., Haq et al. 2019; Aghaie and Tafreshi 2020; Liu et al. 2023), the *HEAT STRESS TRANSCRIPTION FACTOR 6A* (*A6B*), with relevant function in drought stress and rehydration, and related to ABA signaling and ABA‐mediated heat response (e.g., Huang et al. 2016; Singh et al. 2018; Shu et al. 2024), a low‐affinity nitrate transporter (*NRT1.1*), which has been related to nitrate uptake under stress, to differences in nitrogen‐use efficiency and differences in root architecture, to the activity of aquaporins mediating urea uptake (e.g., Renau‐Morata et al. 2021; Hua et al. 2025), and to potassium ion translocation (Martínez‐Martínez et al. 2024), the *PLASMA MEMBRANE INTRINSIC PROTEIN 2.9*, having relevant impact on regulation of leaf and root hydraulic conductance under drought, and related to ABA‐mediated response and to elevated CO_2_ response (*PIP2.9*; e.g., Fang et al. 2019; Li et al. 2021), and the *INDOLE‐3‐ACETIC ACID INDUCIBLE 12* (*IAA12*), a gene of the Aux/*IAA* family with a significant role in abiotic stress tolerance and root development, and key in the auxin signaling transduction (e.g., Audran‐Delalande et al. 2012; Zhang et al. 2021; Zhuang et al. 2025).

Quantification by RT‐qPCR of the aforementioned genes (Figure 7) agreed with the results of the GO Enrichment Analyses, consistently denoting a differential impact of *HSP20* and *HSP70* in the drought stress response in RAM (up to 29‐fold increase in leaves and 9.2‐fold increase in roots). This behaviour in RAM can be coupled to a water conservation strategy at the root level, as denoted by the maximum response to WS in the plasma membrane intrinsic aquaporin (*PIP2.9*) across the studied genotypes, as well as to the maximum response to WS in *SlA6B* in the leaf. As indicated above, this has been related to drought stress and rehydration and to ABA‐dependent responses to face stress (Huang et al. 2016; Singh et al. 2018; Shu et al. 2024). Moreover, quantification analyses highlighted a particularly elevated expression of *PIP2.9* in LUC irrespective of the water treatment, consistent with aquaporin up‐regulation observed in this landrace in previous studies (Landi et al. 2023), suggesting a constitutive response, and a particularly low expression of the *IAA12* in AC under WS as compared to the remaining genotypes (Figure 7). Altogether, results show a clear genotype‐dependent and tissue‐dependent transcriptional response to water stress across the studied genotypes.

### 4.3 Divergent Molecular Responses to WS Between the Mediterranean Tomato Landraces

Notable differences were observed in the response to WS between LUC and RAM (Figures 5 and 6). In roots, LUC showed overexpression of salt transmembrane transporter activity, while RAM displayed down‐regulation; RAM up‐regulated response to salt stress, but LUC did not. This disparity, combined with RAM's up‐regulation of water transmembrane activity, suggests contrasting adaptive strategies in which RAM appears to prioritize water regulation mechanisms, while LUC may specialize in mitigating osmotic stress at the root level. This divergence could reflect their independent adaptation to different regions within the Mediterranean basin.

In leaves, and according to the divergent leaf transcriptomic profile, RAM uniquely exhibited a significant impact in pathways related to heat stress, including response to high‐light intensity and heat‐shock protein binding. This fact suggests a specific mechanism to regulate increased leaf temperature and manage excess electrons resulting from reduced stomatal conductance under stress. Notably, while LUC activated salt response pathways in leaves, RAM did not. Additionally, both landraces up‐regulated aquaporins in leaves but with distinct gene expression patterns, reflecting different mechanisms for maintaining water balance (Table S2). In roots, LUC consistently up‐regulated multiple aquaporins, supporting previous findings on its water management strategies, whereas RAM showed minimal aquaporin activation (Table S3). Finally, and in contrast to the remaining accessions, RAM's response to drought was in part in both leaf and root tissues, with up to three GO terms up‐regulated, namely Response to heat (GO:0009408), *HSP* binding (GO:0031072), and Water transmembrane transporter activity (GO:0005372). This is indeed another relevant difference between the landraces, reinforcing the observation that each landrace may adopt a different strategy to cope with drought stress.

## 5 Concluding Remarks

Overall, this study provides valuable insights into how different tomato genotypes respond to water stress and supports previous research on plant physiology, demonstrating the greater drought resilience of Mediterranean landraces. Findings highlight the diversity of molecular responses to water stress in tomato, with differences also within landraces and within non‐tolerant genotypes, and involving tissue‐specific responses. Besides the genetic background, this variability showed a relevant environmental effect, suggesting that stress intensity is also a prime factor in defining each genotype's response, which might explain heterogeneous results in the literature. The response to water stress in non‐tolerant genotypes reflected a general metabolic regulation and the activation of plant defense pathways. In contrast, the adaptation of landraces is highlighted by a specific response, including pathways related to water, temperature, and osmotic stresses. The response was also variable between landraces. RAM exhibited a stronger leaf transcriptomic response to WS, while LUC prioritized root‐based adaptations. Consistent with this, RAM showed the greatest drought‐induced changes in leaf gene expression, aligning with previously observed extensive leaf adaptations in Balearic LSL landraces under drought conditions. These insights could help in breeding programs aimed at enhancing drought tolerance by targeting the specific pathways identified in this study.

## Author Contributions

A.J.‐C., J.G., J.M., and M.À.C. conceived the research. A.J.‐C., L.C., M.F.‐P., and J.M. performed the research and compiled the data. All the authors analyzed part of the data. A.J.‐C. and M.A.C. wrote the original draft, with relevant contributions from J.M. and J.G. All authors edited and revised the final version of the manuscript.

## Funding

This work was supported by Ministerio de Ciencia e Innovación, CEX2020‐000999‐S; Agencia Estatal de Investigación, PID2020‐114165RR‐C21; Ministerio de Ciencia, Innovación y Universidades, PCI2019‐103610, PCI2019‐103706; Govern de les Illes Balears, FPI_045_2021, PDR2020/59 ‐ ITS2017‐006.

## Conflicts of Interest

The authors declare no conflicts of interest.

## Supporting information


**Dataset S1:** Sheet 1: Log fold‐change (LFC) of all statistically significant differentially expressed genes (DEGs; adjusted *p*‐value < 0.1) with Solgenomics cross‐reference in water stress (WS) versus well‐watered (WW) for leaf and root when not accounting for genotype in the model (i.e., considering together all genes that are DEGs in WS vs. WW in at least one of the genotypes). ND stands for not differentially expressed. Sheet 2: Statistically enriched gene ontology (GO) terms (adjusted *p*‐value < 0.05) in WS versus WW conditions, based on DEGs without accounting for the genotype in the model.

**Dataset S2:** Log fold‐change (LFC) of all statistically significant differentially expressed genes (DEGs; adjusted *p*‐value < 0.1) with Solgenomics cross‐reference in water stress vs. well‐watered for Ailsa Craig (AC), Money‐Maker (MM), Lucariello (LUC) and Ramellet (RAM) genotypes in leaf tissue. ND stands for not differentially expressed.

**Dataset S3:** Log fold‐change (LFC) of all statistically significant differentially expressed genes (DEGs; adjusted *p*‐value < 0.1) with Solgenomics cross‐reference in water stress vs. well‐watered for Ailsa Craig (AC), Money‐Maker (MM), Lucariello (LUC) and Ramellet (RAM) genotypes in root tissue. ND stands for not differentially expressed.

**Dataset S4:** Regulation of all statistically enriched Gene Ontology (GO) terms (adjusted *p*‐value < 0.05) in water stress vs. well‐watered conditions, based on differentially expressed genes (DEGs) exclusive to Ailsa Craig (AC), Money‐Maker (MM), Lucariello (LUC) and Ramellet (RAM) genotypes in leaf tissue. NA stands for not enriched.

**Dataset: S5** Regulation of all statistically enriched Gene Ontology (GO) terms (adjusted *p*‐value < 0.05) in water stress vs. well‐watered conditions, based on differentially expressed genes (DEGs) exclusive to Ailsa Craig (AC), Money‐Maker (MM), Lucariello (LUC) and Ramellet (RAM) genotypes in root tissue. NA stands for not enriched.

**Figure S1:** Top: Diagram of the experimental design and detail of growth conditions. Five plants of each genotype were randomized within two separate water irrigation blocks, one that covered 100% of the evapotranspiration (ETP) needs of the plant (well‐watered treatment, WW), and the other was irrigated with only the 50% of the WW water volume (water stress treatment, WS). After 5 weeks of treatment, all plants were used for leaf gas‐exchange measurements and three of each genotype and treatment were used for RNA‐seq and qPCR analyses. Bottom: Comparison of the different genotypes and treatments at the time of sampling.
**Figure S2:**. Linear regression between qPCR and RNA‐seq expression data based on the log2FC of eight gene models (*SlHSP20, SlHSP70, SlNRT1.1* and *SlA6B* for leaf, and *SlHSP20, SlHSP70, SlPIP2.9* and *SlIAA12* for root) for all four genotypes (AC, MM, LUC and RAM). The correlation between qPCR and RNA‐seq expression resulted significant (Pearson correlation, *R* = 0.41, *p*‐value < 0.05).
**Table S1:** List of significant (*p*‐value ≤ 0.05) DEGs between irrigation treatments in leaves related to photosynthesis, where LFC stands for the log Fold Change of each gene when considering all genotypes (negative values denote down‐regulation in WS vs. WW). Gene IDs and descriptions from SolGenomics.
**Table S2:** List of significant (*p*‐value ≤ 0.05) DEGs belonging to biologically relevant GO terms in leaves of ‘Lucariello’ (LUC) and ‘de Ramellet’ (RAM) genotypes in WS conditions compared to WW conditions, where LFC = Log2 Fold Change and ND = Not Differentially Expressed. Gene IDs, descriptions, and categories from SolGenomics annotation.
**Table S3:** List of significant (*p*‐value ≤ 0.05) DEGs belonging to biologically relevant GO terms in roots of ‘Lucariello’ (LUC) and ‘de Ramellet’ (RAM) genotypes in WS conditions compared to WW conditions, where LFC = Log2 Fold Change and ND = Not Differentially Expressed. Gene IDs, descriptions, and categories from SolGenomics annotation.
**Table S4:** Primers used in RT‐qPCR analyses.

## Data Availability

Raw transcript count data can be accessed at Zenodo, https://doi.org/10.5281/zenodo.15040335. Further data will be made available upon request.

## References

[ppl70696-bib-0001] Aghaie, P. , and S. A. H. Tafreshi . 2020. “Central Role of 70‐kDa Heat Shock Protein in Adaptation of Plants to Drought Stress.” Cell Stress and Chaperones 25, no. 6: 1071–1081. 10.1007/s12192-020-01144-7. 32720054 PMC7591640

[ppl70696-bib-0002] Audran‐Delalande, C. , C. Bassa , I. Mila , F. Regad , M. Zouine , and M. Bouzayen . 2012. “Genome‐Wide Identification, Functional Analysis and Expression Profiling of the *Aux/IAA* Gene Family in Tomato.” Plant and Cell Physiology 53, no. 4: 659–672. 10.1093/pcp/pcs022. 22368074

[ppl70696-bib-0003] Bai, Y. , and P. Lindhout . 2007. “Domestication and Breeding of Tomatoes: What Have We Gained and What Can We Gain in the Future?” Annals of Botany 100, no. 5: 1085–1094. 10.1093/aob/mcm150. 17717024 PMC2759208

[ppl70696-bib-0004] Baldoni, E. , P. Bagnaresi , F. Locatelli , M. Mattana , and A. Genga . 2016. “Comparative Leaf and Root Transcriptomic Analysis of Two Rice *Japonica* Cultivars Reveals Major Differences in the Root Early Response to Osmotic Stress.” Rice 9, no. 1: 1–20. 10.1186/s12284-016-0098-1. 27216147 PMC4877341

[ppl70696-bib-0005] Balestrini, R. , L. C. Rosso , P. Veronico , et al. 2019. “Transcriptomic Responses to Water Deficit and Nematode Infection in Mycorrhizal Tomato Roots.” Frontiers in Microbiology 10: 1–17. 10.3389/fmicb.2019.01807. 31456765 PMC6700261

[ppl70696-bib-0006] Benjamini, Y. , and Y. Hochberg . 1995. “Controlling the False Discovery Rate: A Practical and Powerful Approach to Multiple Testing.” Journal of the Royal Statistical Society, Series B: Statistical Methodology 57, no. 1: 289–300. 10.1111/j.2517-6161.1995.tb02031.x.

[ppl70696-bib-0007] Berger, J. , J. Palta , and V. Vadez . 2016. “Review: An Integrated Framework for Crop Adaptation to Dry Environments: Responses to Transient and Terminal Drought.” Plant Science 253: 58–67. 10.1016/j.plantsci.2016.09.007. 27968997

[ppl70696-bib-0008] Bian, Z. , Y. Wang , X. Zhang , et al. 2021. “A Transcriptome Analysis Revealing the New Insight of Green Light on Tomato Plant Growth and Drought Stress Tolerance.” Frontiers in Plant Science 12: 1–15. 10.3389/fpls.2021.649283. PMC8566944 34745154

[ppl70696-bib-0009] Bolger, A. , F. Scossa , M. E. Bolger , et al. 2014. “The Genome of the Stress‐Tolerant Wild Tomato Species *Solanum pennellii* .” Nature Genetics 46, no. 9: 1034–1038. 10.1038/ng.3046. 25064008 PMC7036041

[ppl70696-bib-0010] Bota, J. , M. À. Conesa , J. M. Ochogavia , H. Medrano , D. M. Francis , and J. Cifre . 2014. “Characterization of a Landrace Collection for Tomàtiga de Ramellet ( *Solanum lycopersicum* L.) From the Balearic Islands.” Genetic Resources and Crop Evolution 61, no. 6: 1131–1146. 10.1007/s10722-014-0096-3.

[ppl70696-bib-0011] Cammarano, D. , D. Ronga , I. Di Mola , M. Mori , and M. Parisi . 2020. “Impact of Climate Change on Water and Nitrogen Use Efficiencies of Processing Tomato Cultivated in Italy.” Agricultural Water Management 241: 106336. 10.1016/j.agwat.2020.106336.

[ppl70696-bib-0012] Caramante, M. , Y. Rouphael , and G. Corrado . 2024. “Genetic Diversity Among and Within Tomato ( *Solanum lycopersicum* L.) Landraces Grown in Southern Italy.” Genetic Resources and Crop Evolution 71, no. 1: 157–166. 10.1007/s10722-023-01613-9.

[ppl70696-bib-0013] Casals, J. , M. Martí , A. Rull , and C. Pons . 2021. “Sustainable Transfer of Tomato Landraces to Modern Cropping Systems: The Effects of Environmental Conditions and Management Practices on Long‐Shelf‐Life Tomatoes.” Agronomy 11, no. 3: 533. 10.3390/AGRONOMY11030533.

[ppl70696-bib-0014] Casañas, F. , J. Simó , J. Casals , and J. Prohens . 2017. “Toward an Evolved Concept of Landrace.” Frontiers in Plant Science 8: 145. 10.3389/fpls.2017.00145. 28228769 PMC5296298

[ppl70696-bib-0015] Cebolla‐Cornejo, J. , S. Roselló , and F. Nuez . 2013. “Phenotypic and Genetic Diversity of Spanish Tomato Landraces.” Scientia Horticulturae 162: 150–164. 10.1016/j.scienta.2013.07.044.

[ppl70696-bib-0016] Chen, T. , G. Qin , and S. Tian . 2020. “Regulatory Network of Fruit Ripening: Current Understanding and Future Challenges.” New Phytologist 228, no. 4: 1219–1226. 10.1111/nph.16822. 32729147

[ppl70696-bib-0017] Comas, L. H. , S. R. Becker , V. M. V. Cruz , P. F. Byrne , and D. A. Dierig . 2013. “Root Traits Contributing to Plant Productivity Under Drought.” Frontiers in Plant Science 4: 1–16. 10.3389/fpls.2013.00442. 24204374 PMC3817922

[ppl70696-bib-0018] Conesa, M. À. , M. Fullana‐Pericàs , A. Granell , and J. Galmés . 2020. “Mediterranean Long Shelf‐Life Landraces: An Untapped Genetic Resource for Tomato Improvement.” Frontiers in Plant Science 10: 1–21. 10.3389/fpls.2019.01651. PMC6965163 31998340

[ppl70696-bib-0019] Conesa, M. À. , J. Galmés , J. M. Ochogavía , et al. 2014. “The Postharvest Tomato Fruit Quality of Long Shelf‐Life Mediterranean Landraces Is Substantially Influenced by Irrigation Regimes.” Postharvest Biology and Technology 93: 114–121. 10.1016/j.postharvbio.2014.02.014.

[ppl70696-bib-0020] Conesa, M. À. , C. D. Muir , E. J. Roldán , A. Molins , J. A. Perdomo , and J. Galmés . 2017. “Growth Capacity in Wild Tomatoes and Relatives Correlates With Original Climate in Arid and Semi‐Arid Species.” Environmental and Experimental Botany 141: 181–190. 10.1016/j.envexpbot.2017.04.009.

[ppl70696-bib-0021] COP29 ‐ Mediterranean Experts on Climate and Environmental Change (MedECC) and Union for the Mediterranean . 2024. “Special Reports on Coastal Risks and the WEFE Nexus.” Conference Presentation. Press Conference at UNFCCC COP29, Baku, Azerbaijan. https://www.medecc.org/outputs/unfccc‐cop29‐press‐conference‐medecc‐special‐reports‐on‐coastal‐risks‐and‐the‐wefe‐nexus/.

[ppl70696-bib-0022] Corrado, G. , M. Caramante , P. Piffanelli , and R. Rao . 2014. “Genetic Diversity in Italian Landraces: Implications for the Development of a Core Collection.” Scientia Horticulturae 168: 138–144. 10.1016/j.scienta.2014.01.027.

[ppl70696-bib-0023] Corrales, A. R. , S. G. Nebauer , L. Carrillo , et al. 2014. “Characterization of Tomato Cycling Dof Factors Reveals Conserved and New Functions in the Control of Flowering Time and Abiotic Stress Responses.” Journal of Experimental Botany 65, no. 4: 995–1012. 10.1093/jxb/ert451. 24399177

[ppl70696-bib-0024] Cramer, W. , J. Guiot , M. Fader , et al. 2018. “Climate Change and Interconnected Risks to Sustainable Development in the Mediterranean.” Nature Climate Change 8, no. 11: 972–980. 10.1038/s41558-018-0299-2.

[ppl70696-bib-0025] Diouf, I. , E. Albert , R. Duboscq , et al. 2020. “Integration of QTL, Transcriptome and Polymorphism Studies Reveals Candidate Genes for Water Stress Response in Tomato.” Genes 11: 900. 10.3390/genes11080900. 32784535 PMC7465520

[ppl70696-bib-0026] Easlon, H. M. , and J. H. Richards . 2009. “Drought Response in Self‐Compatible Species of Tomato (Solanaceae).” American Journal of Botany 96, no. 3: 605–611. 10.3732/ajb.0800189. 21628216

[ppl70696-bib-0027] Ercolano, M. R. , A. Sacco , F. Ferriello , et al. 2014. “Patchwork Sequencing of Tomato San Marzano and Vesuviano Varieties Highlights Genome‐Wide Variations.” BMC Genomics 15, no. 1: 138. 10.1186/1471-2164-15-138. 24548308 PMC3936818

[ppl70696-bib-0028] Fang, L. , L. O. A. Abdelhakim , J. N. Hegelund , et al. 2019. “ABA‐Mediated Regulation of Leaf and Root Hydraulic Conductance in Tomato Grown at Elevated CO_2_ Is Associated With Altered Gene Expression of Aquaporins.” Horticulture Research 6: 104. 10.1038/s41438-019-0187-6. 31645959 PMC6804533

[ppl70696-bib-0029] FAOSTAT ‐ Food and Agriculture Organization of the United Nations . 2025. “FAOSTAT Statistical Database.” https://www.fao.org/faostat/en/#data.

[ppl70696-bib-0030] Flexas, J. , J. Bota , J. Galmés , H. Medrano , and M. Ribas‐Carbó . 2006. “Keeping a Positive Carbon Balance Under Adverse Conditions: Responses of Photosynthesis and Respiration to Water Stress.” Physiologia Plantarum 127, no. 3: 343–352. 10.1111/j.1399-3054.2006.00621.x.

[ppl70696-bib-0031] Foolad, M. R. , L. P. Zhang , and P. Subbiah . 2003. “Genetics of Drought Tolerance During Seed Germination in Tomato: Inheritance and QTL Mapping.” Genome 46, no. 4: 536–545. 10.1139/g03-035. 12897861

[ppl70696-bib-0032] Fuerst, A. , S. Shukla , and Z. Boz . 2025. “Field‐to‐Farm Redesign for Tomato Production to Economically Mitigate Climate Change and Improve Water Sustainability.” Science of the Total Environment 985: 179620. 10.1016/j.scitotenv.2025.179620. 40435726

[ppl70696-bib-0033] Fullana‐Pericàs, M. , M. À. Conesa , C. Douthe , H. El Aou‐ouad , M. Ribas‐Carbó , and J. Galmés . 2019. “Tomato Landraces as a Source to Minimize Yield Losses and Improve Fruit Quality Under Water Deficit Conditions.” Agricultural Water Management 223: 105722. 10.1016/j.agwat.2019.105722.

[ppl70696-bib-0034] Galmés, J. , M. À. Conesa , J. M. Ochogavía , et al. 2011. “Physiological and Morphological Adaptations in Relation to Water Use Efficiency in Mediterranean Accessions of Solanum Lycopersicum.” Plant, Cell & Environment 34, no. 2: 245–260. 10.1111/j.1365-3040.2010.02239.x. 20955222

[ppl70696-bib-0035] Galmés, J. , J. M. Ochogavía , J. Gago , E. J. Roldán , J. Cifre , and M. À. Conesa . 2013. “Leaf Responses to Drought Stress in Mediterranean Accessions of *Solanum lycopersicum* : Anatomical Adaptations in Relation to Gas Exchange Parameters.” Plant, Cell and Environment 36, no. 5: 920–935. 10.1111/pce.12022. 23057729

[ppl70696-bib-0036] Gilbert, M. E. , and V. Medina . 2016. “Drought Adaptation Mechanisms Should Guide Experimental Design.” Trends in Plant Science 21, no. 8: 639–647. 10.1016/j.tplants.2016.03.003. 27090148

[ppl70696-bib-0037] Giorio, P. , G. Guida , C. Mistretta , et al. 2018. “Physiological, Biochemical and Molecular Responses to Water Stress and Rehydration in Mediterranean Adapted Tomato Landraces.” Plant Biology 20, no. 6: 995–1004. 10.1111/plb.12891. 30098088

[ppl70696-bib-0038] Guida, G. , M. H. Sellami , C. Mistretta , et al. 2017. “Agronomical, Physiological and Fruit Quality Responses of Two Italian Long‐Storage Tomato Landraces Under Rain‐Fed and Full Irrigation Conditions.” Agricultural Water Management 180: 126–135. 10.1016/j.agwat.2016.11.004.

[ppl70696-bib-0039] Guo, Y. , C. Huang , Y. Xie , F. Song , and X. Zhou . 2010. “A Tomato Glutaredoxin Gene *SLGRX1* Regulates Plant Responses to Oxidative, Drought and Salt Stresses.” Planta 232, no. 6: 1499–1509. 10.1007/s00425-010-1271-1. 20862491

[ppl70696-bib-0040] Gupta, A. , A. Rico‐Medina , and A. I. Caño‐Delgado . 2020. “The Physiology of Plant Responses to Drought.” Science 368, no. 6488: 266–269. 10.1126/science.aaz7614. 32299946

[ppl70696-bib-0041] Haq, S. , A. Khan , M. Ali , et al. 2019. “Heat Shock Proteins: Dynamic Biomolecules to Counter Plant Biotic and Abiotic Stresses.” International Journal of Molecular Sciences 20, no. 21: 1–31. 10.3390/ijms20215321. PMC6862505 31731530

[ppl70696-bib-0042] Hoffman, N. E. , K. Ko , D. Milkowski , and E. Pichersky . 1991. “Isolation and Characterization of Tomato cDNA and Genomic Clones Encoding the Ubiquitin Gene *ubi3* .” Plant Molecular Biology 17, no. 6: 1189–1201. 10.1007/BF00028735. 1657246

[ppl70696-bib-0043] Hossain, M. A. , S. H. Wani , S. Bhattacharjee , D. J. Burritl , and L. S. P. Tran . 2016. “Drought Stress Tolerance in Plants, Vol 1: Physiology and Biochemistry.” Drought Stress Tolerance in Plants, Vol 1: Physiology and Biochemistry 1: 1–526. 10.1007/978-3-319-28899-4.

[ppl70696-bib-0044] Hua, Q. , Y. Feng , L. Zheng , X. Li , K. Li , and H. Xu . 2025. “Overexpression of SlGRF4 Positively Regulates Drought Stress Tolerance in Tomato by Alleviating ROS Damage and Increasing Nitrogen Signaling Pathway.” Plant Science 358: 112568. 10.1016/j.plantsci.2025.112568. 40398564

[ppl70696-bib-0045] Huang, Y. C. , C. Y. Niu , C. R. Yang , and T. L. Jinn . 2016. “The Heat Stress Factor HSFA6b Connects ABA Signaling and ABA‐Mediated Heat Responses.” Plant Physiology 172, no. 2: 1182–1199. 10.1104/pp.16.00860. 27493213 PMC5047099

[ppl70696-bib-0046] Iovieno, P. , P. Punzo , G. Guida , et al. 2016. “Transcriptomic Changes Drive Physiological Responses to Progressive Drought Stress and Rehydration in Tomato.” Frontiers in Plant Science 7: 1–14. 10.3389/fpls.2016.00371. 27066027 PMC4814702

[ppl70696-bib-0047] Kamenetzky, L. , R. Asís , S. Bassi , et al. 2010. “Genomic Analysis of Wild Tomato Introgressions Determining Metabolism‐and Yield‐Associated Traits.” Plant Physiology 152, no. 4: 1772–1786. 10.1104/pp.109.150532. 20118271 PMC2850009

[ppl70696-bib-0048] Karanja, J. K. , M. M. Aslam , Z. Qian , R. Yankey , I. C. Dodd , and X. Weifeng . 2021. “Abscisic Acid Mediates Drought‐Enhanced Rhizosheath Formation in Tomato.” Frontiers in Plant Science 12: 1–13. 10.3389/fpls.2021.658787. PMC8378331 34421937

[ppl70696-bib-0049] Kim, D. , B. Langmead , and S. L. Salzberg . 2015. “HISAT: A Fast Spliced Aligner With Low Memory Requirements.” Nature Methods 12, no. 4: 357–360. 10.1038/nmeth.3317. 25751142 PMC4655817

[ppl70696-bib-0050] Landi, S. , A. De Lillo , R. Nurcato , S. Grillo , and S. Esposito . 2017. “In‐Field Study on Traditional Italian Tomato Landraces: The Constitutive Activation of the ROS Scavenging Machinery Reduces Effects of Drought Stress.” Plant Physiology and Biochemistry 118: 150–160. 10.1016/j.plaphy.2017.06.011. 28633087

[ppl70696-bib-0051] Landi, S. , P. Punzo , R. Nurcato , et al. 2023. “Transcriptomic Landscape of Tomato Traditional Long Shelf‐Life Landraces Under Low Water Regimes.” Plant Physiology and Biochemistry 201: 107877. 10.1016/j.plaphy.2023.107877. 37473675

[ppl70696-bib-0052] Lazaridi, E. , A. Kapazoglou , M. Gerakari , et al. 2024. “Crop Landraces and Indigenous Varieties: A Valuable Source of Genes for Plant Breeding.” Plants 13, no. 6: 1–23. 10.3390/plants13060758. PMC10975389 38592762

[ppl70696-bib-0053] Lazoglou, G. , A. Papadopoulos‐Zachos , P. Georgiades , et al. 2024. “Identification of Climate Change Hotspots in the Mediterranean.” Scientific Reports 14, no. 1: 29817. 10.1038/s41598-024-80139-1. 39616216 PMC11608228

[ppl70696-bib-0054] Li, S. , L. Fang , J. N. Hegelund , and F. Liu . 2021. “Elevated CO_2_ Modulates Plant Hydraulic Conductance Through Regulation of PIPs Under Progressive Soil Drying in Tomato Plants.” Frontiers in Plant Science 12: 666066. 10.3389/fpls.2021.666066. 34168667 PMC8218578

[ppl70696-bib-0055] Liu, H. , C. Yu , H. Li , et al. 2015. “Overexpression of *ShDHN*, a Dehydrin Gene From *Solanum habrochaites* Enhances Tolerance to Multiple Abiotic Stresses in Tomato.” Plant Science 231: 198–211. 10.1016/j.plantsci.2014.12.006. 25576005

[ppl70696-bib-0056] Liu, M. , H. Yu , G. Zhao , Q. Huang , Y. Lu , and B. Ouyang . 2017. “Profiling of Drought‐Responsive microRNA and mRNA in Tomato Using High‐Throughput Sequencing.” BMC Genomics 18, no. 1: 1–18. 10.1186/s12864-017-3869-1. 28651543 PMC5485680

[ppl70696-bib-0057] Liu, M. , G. Zhao , X. Huang , et al. 2023. “Candidate Regulators of Drought Stress in Tomato Revealed by Comparative Transcriptomic and Proteomic Analyses.” Frontiers in Plant Science 14: 1–16. 10.3389/fpls.2023.1282718. PMC10627169 37936934

[ppl70696-bib-0058] Livak, K. J. , and T. D. Schmittgen . 2001. “Analysis of Relative Gene Expression Data Using Real‐Time Quantitative PCR and the 2^−ΔΔCT^ Method.” Methods 25, no. 4: 402–408. 10.1006/meth.2001.1262. 11846609

[ppl70696-bib-0060] Martínez‐Martínez, A. , M. Á. Botella , M. F. García‐Legaz , et al. 2024. “SlNRT1.5 Transporter and the SlSKOR K+ Channel Jointly Contribute to K+ Translocation in Tomato Plants.” Plant Stress 14: 100689. 10.1016/j.stress.2024.100689.

[ppl70696-bib-0061] Meyer, Y. , B. B. Buchanan , F. Vignols , and J. P. Reichheld . 2009. “Thioredoxins and Glutaredoxins: Unifying Elements in Redox Biology.” Annual Review of Genetics 43: 335–367. 10.1146/annurev-genet-102108-134201. 19691428

[ppl70696-bib-0062] Minh‐Thu, P. T. , D. J. Hwang , J. S. Jeon , B. H. Nahm , and Y. K. Kim . 2013. “Transcriptome Analysis of Leaf and Root of Rice Seedling to Acute Dehydration.” Rice 6, no. 1: 1–18. 10.1186/1939-8433-6-38. 24341907 PMC3878681

[ppl70696-bib-0063] Muir, C. D. , M. Conesa , E. J. Roldán , A. Molins , and J. Galmés . 2017. “Weak Coordination Between Leaf Structure and Function Among Closely Related Tomato Species.” New Phytologist 213, no. 4: 1642–1653. 10.1111/nph.14285. 28164333

[ppl70696-bib-0064] Muluneh, M. G. 2021. “Impact of Climate Change on Biodiversity and Food Security: A Global Perspective—A Review Article.” Agriculture & Food Security 10, no. 1: 1–25. 10.1186/s40066-021-00318-5.

[ppl70696-bib-0065] Muñoz‐Espinoza, V. A. , M. F. López‐Climent , J. A. Casaretto , and A. Gómez‐Cadenas . 2015. “Water Stress Responses of Tomato Mutants Impaired in Hormone Biosynthesis Reveal Abscisic Acid, Jasmonic Acid and Salicylic Acid Interactions.” Frontiers in Plant Science 6: 997. 10.3389/fpls.2015.00997. 26635826 PMC4649032

[ppl70696-bib-0066] Patanè, C. , D. Scordia , G. Testa , and S. L. Cosentino . 2016. “Physiological Screening for Drought Tolerance in Mediterranean Long‐Storage Tomato.” Plant Science 249: 25–34. 10.1016/j.plantsci.2016.05.006. 27297987

[ppl70696-bib-0067] Patanè, C. , S. Tringali , and O. Sortino . 2011. “Effects of Deficit Irrigation on Biomass, Yield, Water Productivity and Fruit Quality of Processing Tomato Under Semi‐Arid Mediterranean Climate Conditions.” Scientia Horticulturae 129, no. 4: 590–596. 10.1016/j.scienta.2011.04.030.

[ppl70696-bib-0068] Peralta, I. E. , D. M. Spooner , and S. Knapp . 2008. “The Taxonomy of Tomatoes: A Revision of Wild Tomatoes (Solanum Section Lycopersicon) and Their Outgroup Relatives in Sections Juglandifolium and Lycopersicoides.” Systematic Botany Monographs 84: 1–186.

[ppl70696-bib-0069] Pereira, L. S. , I. Cordery , and I. Iacovides . 2012. “Improved Indicators of Water Use Performance and Productivity for Sustainable Water Conservation and Saving.” Agricultural Water Management 108: 39–51. 10.1016/j.agwat.2011.08.022.

[ppl70696-bib-0070] Pirona, R. , G. Frugis , F. Locatelli , M. Mattana , A. Genga , and E. Baldoni . 2023. “Transcriptomic Analysis Reveals the Gene Regulatory Networks Involved in Leaf and Root Response to Osmotic Stress in Tomato.” Frontiers in Plant Science 14: 1155797. 10.3389/fpls.2023.1155797. 37332696 PMC10272567

[ppl70696-bib-0072] Renau‐Morata, B. , R. V. Molina , E. G. Minguet , et al. 2021. “Integrative Transcriptomic and Metabolomic Analysis at Organ Scale Reveals Gene Modules Involved in the Responses to Suboptimal Nitrogen Supply in Tomato.” Agronomy 11, no. 7: 1320. 10.3390/agronomy11071320.

[ppl70696-bib-0073] Ripoll, J. , L. Urban , and N. Bertin . 2016. “The Potential of the MAGIC TOM Parental Accessions to Explore the Genetic Variability in Tomato Acclimation to Repeated Cycles of Water Deficit and Recovery.” Frontiers in Plant Science 6: 1172. 10.3389/fpls.2015.01172. 26779213 PMC4700940

[ppl70696-bib-0074] Saleem, M. , Q. Fariduddin , and T. Janda . 2021. “Multifaceted Role of Salicylic Acid in Combating Cold Stress in Plants: A Review.” Journal of Plant Growth Regulation 40, no. 2: 464–485. 10.1007/s00344-020-10152-x.

[ppl70696-bib-0075] Shu, J. , L. Zhang , G. Liu , et al. 2024. “Transcriptome Analysis and Metabolic Profiling Reveal the Key Regulatory Pathways in Drought Stress Responses and Recovery in Tomatoes.” International Journal of Molecular Sciences 25, no. 4: 2187. 10.3390/ijms25042187. 38396864 PMC10889177

[ppl70696-bib-0076] Singh, G. , N. K. Sarkar , and A. Grover . 2018. “Mapping of Domains of Heat Stress Transcription Factor OsHsfA6a Responsible for Its Transactivation Activity.” Plant Science 274: 80–90. 10.1016/j.plantsci.2018.05.010. 30080644

[ppl70696-bib-0077] Tamburino, R. , M. Vitale , A. Ruggiero , et al. 2017. “Chloroplast Proteome Response to Drought Stress and Recovery in Tomato (*Solanum lycopersicum* L.).” BMC Plant Biology 17, no. 1: 40. 10.1186/s12870-017-0971-0. 28183294 PMC5301458

[ppl70696-bib-0078] Terzopoulos, P. J. , and P. J. Bebeli . 2008. “DNA and Morphological Diversity of Selected Greek Tomato ( *Solanum lycopersicum* L.) Landraces.” Scientia Horticulturae 116, no. 4: 354–361. 10.1016/j.scienta.2008.02.010.

[ppl70696-bib-0079] The Tomato Genome Consortium . 2012. “The Tomato Genome Sequence Provides Insights Into Fleshy Fruit Evolution.” Nature 485, no. 7400: 635–641. 10.1038/nature11119. 22660326 PMC3378239

[ppl70696-bib-0080] Tranchida‐Lombardo, V. , R. Aiese Cigliano , I. Anzar , et al. 2018. “Whole‐Genome Re‐Sequencing of Two Italian Tomato Landraces Reveals Sequence Variations in Genes Associated With Stress Tolerance, Fruit Quality and Long Shelf‐Life Traits.” DNA Research 25, no. 2: 149–160. 10.1093/dnares/dsx045. 29149280 PMC5909465

[ppl70696-bib-0081] UNEP ‐ United Nations Environment Programme . 2021. Climate Change in the Mediterranean. UNEP/MAP. https://www.unep.org/unepmap/resources/factsheets/climate‐change.

[ppl70696-bib-0082] Veronico, P. , L. C. Rosso , M. T. Melillo , et al. 2022. “Water Stress Differentially Modulates the Expression of Tomato Cell Wall Metabolism‐Related Genes in Meloidogyne Incognita Feeding Sites.” Frontiers in Plant Science 13: 817185. 10.3389/fpls.2022.817185. 35498686 PMC9051518

[ppl70696-bib-0083] Villena, J. , C. Moreno , S. Roselló , J. Cebolla‐Cornejo , and M. M. Moreno . 2023. “Dissecting a Vegetable Landrace: Components of Variation in Spanish ‘Moruno’ Tomatoes as a Case Studio.” Scientia Horticulturae 318: 112128. 10.1016/j.scienta.2023.112128.

[ppl70696-bib-0084] Xu, H. , P. Liu , C. Wang , et al. 2022. “Transcriptional Networks Regulating Suberin and Lignin in Endodermis Link Development and ABA Response.” Plant Physiology 190, no. 2: 1165–1181. 10.1093/plphys/kiac298. 35781829 PMC9516719

[ppl70696-bib-0085] Yamasaki, Y. , G. Koehler , B. J. Blacklock , and S. K. Randall . 2013. “Dehydrin Expression in Soybean.” Plant Physiology and Biochemistry 70: 213–220. 10.1016/j.plaphy.2013.05.013. 23792826

[ppl70696-bib-0086] Yang, L. , S. Bu , S. Zhao , et al. 2022. “Transcriptome and Physiological Analysis of Increase in Drought Stress Tolerance by Melatonin in Tomato.” PLoS One 17: e0267594. 10.1371/journal.pone.0267594. 35580092 PMC9113596

[ppl70696-bib-0087] Zeven, A. C. 1998. “Landraces: A Review of Definitions and Classifications.” Euphytica 104: 127–139. 10.1023/A:1018683119237.

[ppl70696-bib-0088] Zhang, M. M. , H. K. Zhang , J. F. Zhai , X. S. Zhang , Y. L. Sang , and Z. J. Cheng . 2021. “ARF4 Regulates Shoot Regeneration Through Coordination With ARF5 and IAA12.” Plant Cell Reports 40: 315–325. 10.1007/s00299-020-02633-w. 33180161

[ppl70696-bib-0089] Zhang, Z. , B. Cao , N. Li , Z. Chen , and K. Xu . 2019. “Comparative Transcriptome Analysis of the Regulation of ABA Signaling Genes in Different Rootstock Grafted Tomato Seedlings Under Drought Stress.” Environmental and Experimental Botany 166: 103814. 10.1016/j.envexpbot.2019.103814.

[ppl70696-bib-0090] Zhou, R. , X. Yu , T. Zhao , C. O. Ottosen , E. Rosenqvist , and Z. Wu . 2019. “Physiological Analysis and Transcriptome Sequencing Reveal the Effects of Combined Cold and Drought on Tomato Leaf.” BMC Plant Biology 19, no. 1: 377. 10.1186/s12870-019-1982-9. 31455231 PMC6712725

[ppl70696-bib-0091] Zhu, J. K. 2016. “Abiotic Stress Signaling and Responses in Plants.” Cell 167, no. 2: 313–324. 10.1016/j.cell.2016.08.029. 27716505 PMC5104190

[ppl70696-bib-0092] Zhuang, Z. , J. Bian , Z. Ren , W. Ta , and Y. Peng . 2025. “Plant *Aux/IAA* Gene Family: Significance in Growth, Development and Stress Responses.” Agronomy 15, no. 5: 1228. 10.3390/agronomy15051228.

[ppl70696-bib-0093] Živanović, B. , S. Milić Komić , N. Nikolić , et al. 2021. “Differential Response of Two Tomato Genotypes, Wild Type cv. Ailsa Craig and Its ABA‐Deficient Mutant Flacca to Short‐Termed Drought Cycles.” Plants 10, no. 11: 2308. 10.3390/plants10112308. 34834671 PMC8617711

