# Supplementary material for: Mediterranean Tomato Landraces Exhibit Genotype‐Specific Transcriptomic Responses to Water Stress
Source: Physiol Plant. 2025 Dec 18;177(6):e70696. doi: 10.1111/ppl.70696 (PMC12715300; doi:10.1111/ppl.70696)
Supplement: Supplementary file 6 — Figure S1: Top: Diagram of the experimental design and detail of growth conditions. Five plants of each genotype were randomized within two separate water irrigation blocks, one that covered 100% of the evapotranspiration (ETP) needs of the plant (well‐watered treatment, WW), and the other was irrigated with only the 50% of the WW water volume (water stress treatment, WS). After 5 weeks of treatment, all plants were used for leaf gas‐exchange measurements and three of each genotype and treatment were used for RNA‐seq and qPCR analyses. Bottom: Comparison of the different genotypes and treatments at the time of sampling. Figure S2:. Linear regression between qPCR and RNA‐seq expression data based on the log2FC of eight gene models (SlHSP20, SlHSP70, SlNRT1.1 and SlA6B for leaf, and SlHSP20, SlHSP70, SlPIP2.9 and SlIAA12 for root) for all four genotypes (AC, MM, LUC and RAM). The correlation between qPCR and RNA‐seq expression resulted significant (Pearson correlation, R = 0.41, p‐value < 0.05). Table S1: List of significant (p‐value ≤ 0.05) DEGs between irrigation treatments in leaves related to photosynthesis, where LFC stands for the log Fold Change of each gene when considering all genotypes (negative values denote down‐regulation in WS vs. WW). Gene IDs and descriptions from SolGenomics. Table S2: List of significant (p‐value ≤ 0.05) DEGs belonging to biologically relevant GO terms in leaves of ‘Lucariello’ (LUC) and ‘de Ramellet’ (RAM) genotypes in WS conditions compared to WW conditions, where LFC = Log2 Fold Change and ND = Not Differentially Expressed. Gene IDs, descriptions, and categories from SolGenomics annotation. Table S3: List of significant (p‐value ≤ 0.05) DEGs belonging to biologically relevant GO terms in roots of ‘Lucariello’ (LUC) and ‘de Ramellet’ (RAM) genotypes in WS conditions compared to WW conditions, where LFC = Log2 Fold Change and ND = Not Differentially Expressed. Gene IDs, descriptions, and categories from SolGenomics annotation. Table [file PPL-177-e70696-s003.pdf]

Mediterranean Tomato Landraces Exhibit Genotype-Specific Transcriptomic Responses to Water Stress

Juan-Cabot, A.; Carrillo, L.; Fullana-Pericàs, M.; Galmés, J.; Medina, J.; Conesa, M.À.

Supplementary figures

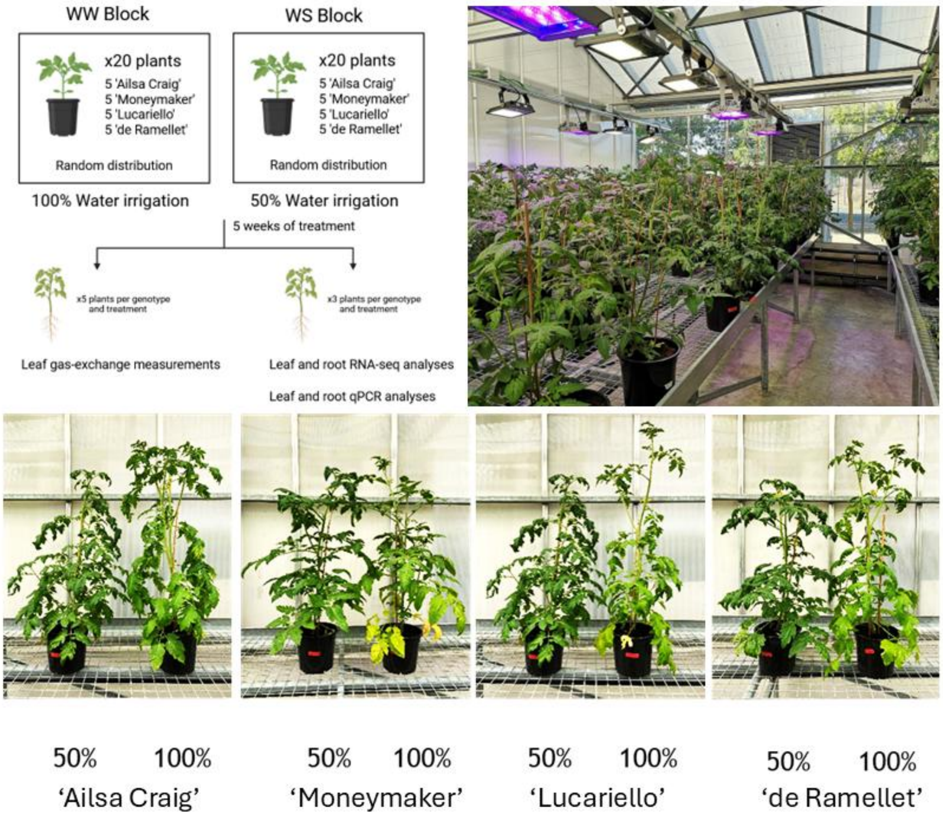

Figure S1. Top: Diagram of the experimental design and detail of growth conditions. Five plants of each genotype were randomized within two separate water irrigation blocks, one that covered 100% of the evapotranspiration (ETP) needs of the plant (well-watered treatment, WW), and the other was irrigated with only the 50% of the WW water volume (water stress treatment, WS). After five weeks of treatment, all plants were used for leaf gas-exchange measurements and three of each genotype and treatment were used for RNA-seq and qPCR analyses. Bottom: Comparison of the different genotypes and treatments at the time of sampling.

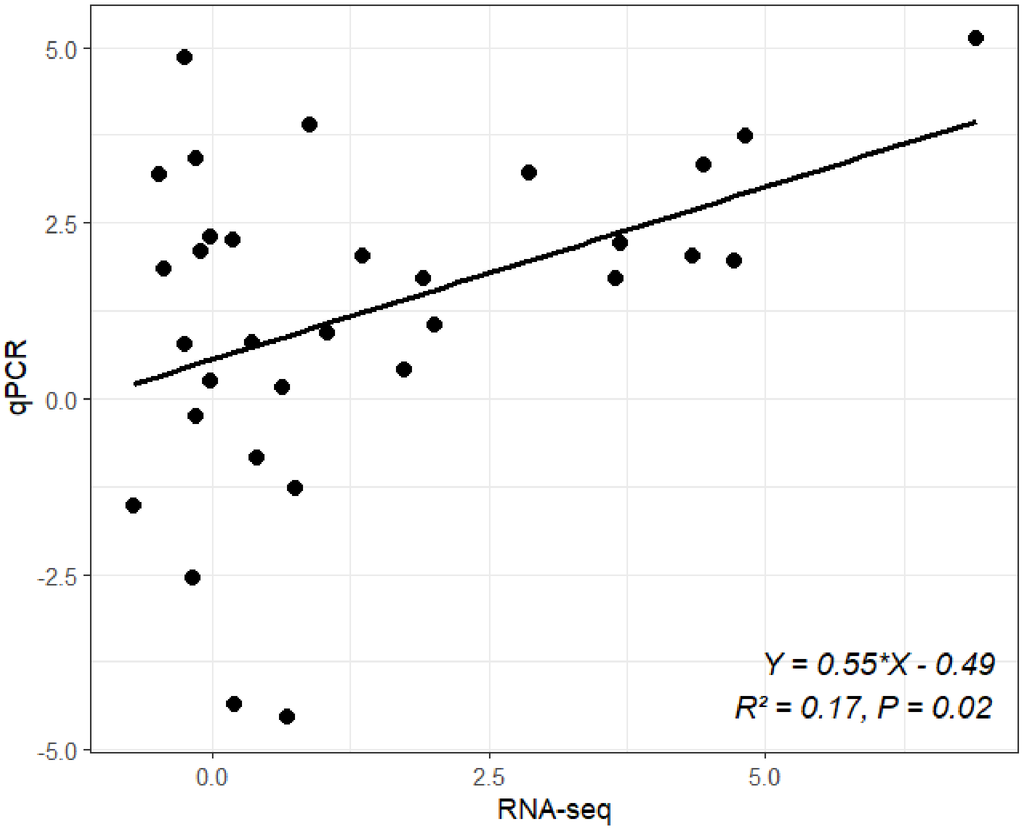

Figure S2. Linear regression between qPCR and RNA-seq expression data based on the log2FC of eight gene models (*SlHSP20, SlHSP70, SlNRT1.1* and *SlA6B* for leaf, and *SlHSP20, SlHSP70, SlPIP2.9* and *SlIAA12* for root) for all four genotypes (AC, MM, LUC and RAM). The correlation between qPCR and RNA-seq expression resulted significant (Pearson correlation, R = 0.41, *p*-value < 0.05).

Supplementary tables

Table S1. List of significant (*p*-value ≤ 0.05) DEGs between irrigation treatments in leaves related to photosynthesis, where LFC stands for the log Fold Change of each gene when considering all genotypes (negative values denote down-regulation in WS vs. WW). Gene IDs and descriptions from SolGenomics.

Gene ID	LFC	Description
Solyc02g079950.3	-2.05	photosystem II oxygen-evolving complex protein 3
Solyc06g054260.1	-2.29	photosystem 1 reaction center protein subunit 2
Solyc04g082010.1	-2.25	pre-plastocyanin *X13934*
Solyc07g044860.3	-1.99	psbXphotosystem II 23 kDa protein
Solyc02g065400.3	-1.90	33kDa precursor protein of oxygen-evolving complex
Solyc07g066310.3	-1.15	photosystem II polypeptide
Solyc12g005630.2	-1.06	Cytochrome b6-f complex iron-sulfur subunit
Solyc12g044280.2	-3.16	Photosystem I reaction center subunit VI, chloroplastic
Solyc02g069460.3	-1.76	Photosystem I reaction center subunit III
Solyc05g007780.3	-1.82	Oxygen evolving enhancer protein 3
Solyc09g064500.3	-1.78	Photosystem II reaction center psb28 protein
Solyc06g066640.3	-4.16	Photosystem I reaction center subunit VI-1, chloroplastic
Solyc02g080540.1	-1.19	ATP synthase gamma chain
Solyc12g056830.1	-1.88	ATP synthase delta subunit
Solyc12g005060.1	0.57	ATP synthase gamma chain
Solyc03g114930.3	-1.73	Photosystem II oxygen evolving complex protein PsbP
Solyc06g060340.3	-1.32	Chloroplast photosystem II-associated protein
Solyc06g065990.1	-1.67	ATP synthase subunit B / B
Solyc07g066150.1	-2.25	Photosystem I reaction center subunit V
Solyc03g120640.3	-1.66	Photosystem I reaction center subunit VI-1, chloroplastic
Solyc06g082940.3	-1.59	Photosystem I reaction center subunit XI
Solyc06g082950.3	-2.49	Photosystem I reaction center subunit XI
Solyc10g075160.1	-1.21	Ferredoxin I
Solyc08g077050.3	-1.40	Ferredoxin family protein
Solyc06g083680.3	-1.76	Photosystem I reaction center subunit IV A
Solyc09g063130.3	-1.90	Photosystem I reaction center subunit IV A
Solyc08g013670.3	-1.97	Photosystem I reaction center subunit

Table S2. List of significant (*p*-value ≤ 0 .05) DEGs belonging to biologically relevant GO terms in leaves of 'Lucariello' (LUC) and 'de Ramellet' (RAM) genotypes in WS conditions compared to WW conditions, where LFC = Log2 Fold Change and ND = Not Differentially Expressed. Gene IDs, descriptions, and categories from SolGenomics annotation

Gene	LFC RAM	LFC LUC	Description	Category	GO Term
Solyc01g079170.3	3.46	2.27	Galactinol synthase (AHRD V1 ***- D6QSF5_BRANA)	Heat stress response	Response to heat (GO:0009408)
Solyc01g099620.3	6.49	ND	Respiratory burst oxidase-like protein (AHRD V1 **** C1IHQ9_9ROSI)		
Solyc02g079930.3	6.34	2.86	phosphosulfolactate synthase		
Solyc02g093600.3	6.98	3.61	class I heat shock protein (AHRD V1 ***- Q69BI7_CARPA)		
Solyc03g082420.3	6.45	ND	Heat shock protein (AHRD V1 ***- A9QVH3_9FABA)		
Solyc03g117630.1	6.71	2.14	heat shock protein (AHRD V1 ***- B2D2G5_CAPSN)		
Solyc03g123540.3	6.94	2.63	class II heat shock protein (AHRD V1 ***- B6U175_MAIZE)		
Solyc04g014480.3	2.92	ND	class I heat shock protein 3 (AHRD V1 ***- B6TQD6_MAIZE) Hsp20		
Solyc04g072250.3	10.05	ND	class I heat shock protein 1 (AHRD V1 ***- B6TTC8_MAIZE)		
Solyc04g078770.3	ND	2.14	Heat stress transcription factor (AHRD V1 *-*- D4QAU8_CARPA)		
Solyc06g076020.3	3.6	ND	heat shock protein 70 kD		
Solyc07g040680.3	3.38	ND	Heat stress transcription factor A3		
Solyc08g062340.3	4.68	2.57	class II heat shock protein (AHRD V1 ***- B6T339_MAIZE)		
Solyc08g062450.1	7.72	4.12	class II heat shock protein (AHRD V1 ***- B6T339_MAIZE)		
Solyc09g075950.1	3.6	ND	Heat shock protein 1 (AHRD V1 ***- B6SXY0_MAIZE)		
Solyc11g020040.2	3.52	ND	heat shock protein 70		
Solyc11g020330.1	9.31	4.8	leer-sHSP small heat shock protein		
Solyc11g066100.2	3.99	ND	heat shock protein (AHRD V1 ***- B2D2G5_CAPSN)		
Solyc11g071830.2	3.19	ND	Chaperone protein dnaj (AHRD V1 ***- Q17AX7_AEDAE)		
Solyc01g102960.3	7.05	4.46	class IV heat shock protein (AHRD V1 ***- B6T3F5_MAIZE) Hsp70		Response to temperature stimulus (GO:0009266)
Solyc04g073990.3	3.94	ND	annexin p34		Response to stress (GO:0006950)
Solyc01g065700.3	ND	3.37	Integrin-linked kinase-associated serine/threonine phosphatase 2C (AHRD V1 **** ILKAP_BOVIN)	Drought stress response	Drought recovery (GO:0009819)
Solyc01g109920.2	8.14	ND	Dehydrin (AHRD V1 *-*- Q6YL18_BRAJU)		Response to water

Solyc02g084850.3	9.97	5.04	Unknown Protein (AHRD V1)		deprivation (GO:0009414)
Solyc04g071610.3	3.43	ND	Abscicic acid stress ripening 1		
Solyc05g007300.3	ND	1.82	Receptor expression-enhancing protein 5 (AHRD V1 *--* C3KJ86_ANOFI)		
Solyc08g007430.2	6.91	4.35	NIT2		
Solyc09g075550.3	ND	1.99	Cellulose synthase-like D6 (AHRD V1 **** Q09HT5_PHYPA)		
Solyc01g067460.2	4.25	ND	Glutaredoxin family protein (AHRD V1 ***- D7MAF1_ARALY)	REDOX reactions	ND
Solyc04g011790.1	ND	9.1	Glutaredoxin (AHRD V1 **** B9I9V9_POPTR)		
Solyc04g011800.1	ND	8.73	Glutaredoxin (AHRD V1 **** B9I9V9_POPTR)		
Solyc04g011810.1	ND	7.96	Glutaredoxin (AHRD V1 ***- B9I9V9_POPTR)		
Solyc04g011830.1	ND	9.12	Glutaredoxin (AHRD V1 **** B9IBJ4_POPTR)		
Solyc04g011840.1	7.21	8.51	Glutaredoxin (AHRD V1 **** B9I9V9_POPTR)		
Solyc04g011850.1	ND	8.29	Glutaredoxin (AHRD V1 ***- B9I9V9_POPTR)		
Solyc04g011860.1	5.71	6.59	Glutaredoxin (AHRD V1 ***- B9I9V9_POPTR)		
Solyc04g053110.1	4.67	ND	Glutaredoxin (AHRD V1 **** B9I9V9_POPTR)		
Solyc05g006870.3	4.05	ND	Thioredoxin H (AHRD V1 ***- Q4U0W0_NICAL)		
Solyc06g054570.1	4.74	ND	Glutaredoxin family protein (AHRD V1 ***- D7LYE9_ARALY)		
Solyc03g019820.3	11.56	ND	tonoplast intrinsic protein 3.2	Transmembrane transport	Transmembrane transport (GO:0055085)
Solyc06g072130.3	13.24	ND	tonoplast intrinsic protein 3.1		
Solyc01g094690.3	ND	2.04	plasma membrane intrinsic protein 1.2		
Solyc02g068080.3	3.85	1.81	Voltage-gated chloride channel (AHRD V1 **** Q96325_ARATH)		
Solyc03g112100.3	ND	2.39	Component of high affinity nitrate transporter (AHRD V1 **-* D2KTV0_LOTJA)		
Solyc08g008050.3	ND	2.85	plasma membrane intrinsic protein 1.1		
Solyc10g084120.2	3.8	6.05	plasma membrane intrinsic protein 2.5		
Solyc01g094960.3	-0.83	-0.44	mitogen-activated protein kinase 5	MAP Kinase Pathway	MAP kinase activity (GO:0004707)
Solyc03g019850.3	ND	-0.5	MAP kinase kinase 5		
Solyc04g007710.3	-0.76	ND	mitogen-activated protein kinase 14		
Solyc04g080730.3	-0.64	-0.58	mitogen-activated protein kinase 9		
Solyc07g056350.3	ND	-0.51	mitogen-activated protein kinase 12		

Table S3. List of significant (*p*-value ≤0.05) DEGs belonging to biologically relevant GO terms in roots of ‘Lucariello’ (LUC) and ‘de Ramellet’ (RAM) genotypes in WS conditions compared to WW conditions, where LFC = Log2 Fold Change and ND = Not Differentially Expressed. Gene IDs, descriptions, and categories from SolGenomics annotation.

Gene	LFC RAM	LFC LUC	Description	Category	GO Term
Solyc02g079930.3	3.91	3.09	phosphosulfolactate synthase	Heat stress response	Response to heat (GO:0009408)
Solyc02g093600.3	3.08	2.92	class I heat shock protein (AHRD V1 ***- Q69BI7_CARPA)		
Solyc03g082420.3	3.62	ND	Heat shock protein (AHRD V1 ***- A9QVH3_9FABA) Hsp20		
Solyc03g097120.3	1.98	1.35	Heat stress transcription factor A3 (AHRD V1 *-** D1M7W9_SOLLC)		
Solyc03g117630.1	2.78	2.31	heat shock protein (AHRD V1 ***- B2D2G5_CAPSN) Hsp70		
Solyc03g123540.3	2.61	3.35	class II heat shock protein (AHRD V1 ***- B6U175_MAIZE) Hsp20		
Solyc04g014480.3	1.98	ND	class I heat shock protein 3		
Solyc04g072250.3	5.98	3.45	class I heat shock protein 1 (AHRD V1 ***- B6TTC8_MAIZE)		
Solyc08g062340.3	2.84	ND	class II heat shock protein (AHRD V1 ***- B6T339_MAIZE) Hsp20		
Solyc08g062340.3	2.84	ND	class II heat shock protein (AHRD V1 ***- B6T339_MAIZE)		
Solyc08g062450.1	5.01	2.29	class II heat shock protein (AHRD V1 ***- B6T339_MAIZE) Hsp20		
Solyc11g020330.1	3.27	ND	leer-sHSP small heat shock protein		
Solyc11g071830.2	2.34	1.72	Chaperone protein dnaj (AHRD V1 ***- Q17AX7_AEDAE)		
Solyc12g007070.2	2.08	1.58	Heat stress transcription factor A3 (AHRD V1 ***- D1M7W9_SOLLC)		Cellular response to heat (GO:0034605)
Solyc07g040680.3	4.56	3.62	Heat stress transcription factor A3		
Solyc06g076020.3	2.5	ND	heat shock protein 70 kD		Heat shock protein binding (GO:0031072)
Solyc11g020040.2	1.95	ND	heat shock protein 70		
Solyc11g066100.2	3.22	2.56	heat shock protein (AHRD V1 ***- B2D2G5_CAPSN) Hsp70		
Solyc01g102960.3	3.82	2.86	class IV heat shock protein (AHRD V1 ***- B6T3F5_MAIZE)		Response to temperature stimulus (GO:0009266)
Solyc04g073990.3	ND	0.78	annexin p34		Response to stress (GO:0006950)
GO:0000978	2.18	1.48	Heat stress transcription factor		DNA-binding transcription factor activity (GO:0043621)

Solyc01g109920.2	ND	2.9	Dehydrin (AHRD V1 *-*- Q6YL18_BRAJU)	Drought stress response	Response to water deprivation (GO:0009414) ND
Solyc02g080120.2	2.61	ND	Gibberellin 2-beta-dioxygenase 7		
Solyc02g084850.3	ND	2.91	Unknown Protein (AHRD V1)		
Solyc08g007430.2	ND	6.75	NIT2		
Solyc08g078950.3	ND	1.08	NIT1		
Solyc12g056120.1	ND	0.67	6-phosphogluconate dehydrogenase decarboxylating		
Solyc09g074910.1	4.17	ND	TspO and MBR like protein (AHRD V1 **-- D2RRW7_HALTV)		Response to osmotic stress (GO:0006970)
Solyc03g007430.3	ND	0.72	Mitochondrial carrier family (AHRD V1 ***- C1E2Y0_9CHLO)		Stress response to metal ion (GO:0097501)
Solyc03g112440.1	3.96	1.98	Oleosin (AHRD V1 ***- O04925_SESIN)	Lipid storage	Lipid storage (GO:0019915)
Solyc06g034040.1	4.96	3.93	Oleosin (AHRD V1 ***- A5JVA7_FICAW)		
Solyc06g060840.1	ND	6.78	Oleosin (AHRD V1 ***- Q2TM28_COFCA)		
Solyc06g069260.1	4.21	4.26	Oleosin Bn-V (AHRD V1 ***- B6TL84_MAIZE)		
Solyc08g078160.3	5.77	2.94	Oleosin (AHRD V1 ***- Q2TM28_COFCA)		
Solyc12g010920.2	7.01	2.76	Oleosin (AHRD V1 ***- Q8LGL8_OLEEU)		
Solyc01g060070.3	2.08	ND	Pore protein homolog (AHRD V1 **-- Q3EA11_ARATH)	Transmembrane transport	Transmembrane transport (GO:0055085)
Solyc02g068080.3	ND	1.3	Voltage-gated chloride channel (AHRD V1 **** Q96325_ARATH)		
Solyc02g094060.3	ND	3.01	Voltage-gated chloride channel (AHRD V1 **** Q96325_ARATH)		
Solyc03g013160.3	2.18	ND	Amino acid transporter family protein (AHRD V1 **** D7M7A7_ARALY)		
Solyc03g013440.3	2.89	ND	Amino acid transporter family protein (AHRD V1 **** D7LM67_ARALY)		
Solyc03g019820.3	ND	4.44	tonoplast intrinsic protein 3.2		
Solyc03g032090.1	2.16	ND	Amino acid transporter family protein (AHRD V1 **** D7LI10_ARALY)		
Solyc03g078150.3	3.12	ND	Amino acid transporter family protein (AHRD V1 **** D7LGK0_ARALY)		
Solyc06g050790.3	1.04	ND	Amino acid transporter (AHRD V1 **** B9HYI5_POPTR)		
Solyc06g050800.3	3.68	ND	Amino acid transporter (AHRD V1 **** B9HYI5_POPTR)		
Solyc06g010250.3	ND	1.73	Nitrate transporter (AHRD V1 **** A5JUX2_9ROSI)		
Solyc06g011350.3	ND	1.3	plasma membrane intrinsic protein 2.4		
Solyc06g068720.3	1.02	ND	Calcium-binding mitochondrial carrier protein		

			SCaMC-1 (AHRD V1 **** B5X2X8_SALSA)
Solyc06g071890.3	1.56	ND	Brain protein 44-like protein (AHRD V1 ***- B6TQE5_MAIZE)
Solyc06g072130.3	ND	4.26	tonoplast intrinsic protein 3.1
Solyc06g074820.3	ND	0.75	tonoplast intrinsic protein 1.1
Solyc06g075650.3	ND	1.51	tonoplast intrinsic protein 1.2
Solyc08g008050.3	ND	1.28	plasma membrane intrinsic protein 1.1
Solyc08g081190.3	ND	0.85	plasma membrane intrinsic protein 1.5
Solyc10g084120.2	ND	2.01	plasma membrane intrinsic protein 2.5
Solyc11g012360.2	1.29	ND	Sodium-dependent dicarboxylate transporter (AHRD V1 ***- O35055_RAT)
Solyc11g013310.2	0.94	ND	SlLAX3 Auxin transporter
Solyc11g069760.1	ND	4.21	High affinity nitrate transporter protein (AHRD V1 ***- Q84MZ8_TOBAC)
Solyc12g056220.2	ND	0.58	plasma membrane intrinsic protein 1.3
Solyc12g088190.2	2.45	ND	Amino acid permease 6 (AHRD V1 **** Q7Y076_BRANA)

Table S4. Primers used in RT-qPCR analyses.

Gene	Gene ID	Primer sequences
SlPIP2.9	*Solyc10g055630*	GCAATGGCAGCAGCAATATACCA<br>CGAAAGAGAATAGACCACCA
SlNRT1.1	*Solyc08g007430*	TTTTAGGTGTTGAAGCTGTGGAGAG<br>GCGATGTATAGGACCATGAGTTGTT
SlHSP20	*Solyc06g076570*	AAGATTTAGACTTCCGGAGAATGC<br>GCCCATACAAGGATACAATAAATTCACAT
SlHSP70	*Solyc04g011440*	GCAGCATGATCGAGTTGAGA<br>TTGAAAGGCCAAAGCTTCAT
SlA6B	*Solyc06g053960*	AGCAAAAGCTTCAAGGGACA<br>ATCAATTGGCCTCCTCCTCT
SlIAA12	*Solyc09g064530*	TCTTAAGACCCAGCACGACT<br>ATAGGCTTAGTCCCCGGTCT
